# Rational design of hyperstable antibacterial peptides for food preservation

**DOI:** 10.1038/s41538-021-00109-z

**Published:** 2021-09-01

**Authors:** Yashavantha L. Vishweshwaraiah, Abhishek Acharya, Vinayak Hegde, Balaji Prakash

**Affiliations:** 1 Department of Molecular Nutrition, CSIR-Central Food Technological Research Institute, Mysore, India; 2 Academy of Scientific and Innovative Research, Ghaziabad, Uttar Pradesh India; 3 Division of Biological and Life Sciences, School of Arts and Sciences, Ahmedabad University, Ahmedabad, Gujarat India

**Keywords:** Antimicrobials, Peptides

## Abstract

We describe the design of peptides with properties like thermostability, pH stability, and antibacterial activity against a few bacterial food pathogens. Insights obtained from classical structure-function analysis of natural peptides and their mutants through antimicrobial and enzymatic assays are used to rationally develop a set of peptides. pH and thermostability assays were performed to demonstrate robust antimicrobial activity post-treatment with high temperatures and at wide pH ranges. We have also investigated the mode of action of these hyperstable peptides using membrane permeability assays, electron microscopy, and molecular dynamics simulations. Notably, through mutational studies, we show that these peptides elicit their antibacterial action via both membrane destabilization and inhibition of intracellular trypsin—the two functions attributable to separate peptide segments. Finally, toxicity studies and food preservation assays demonstrate the safety and efficacy of the designed peptides for food preservation. Overall, the study provides a general ‘blueprint’ for the development of stable antimicrobial peptides (AMPs). Insights obtained from this work may also be combined with combinatorial methods in high-throughput studies for future development of antimicrobials for various applications.

## Introduction

Microbial action is the most common cause of spoilage and one of most concern as it is also associated with the risks of food poisoning and food-borne illnesses. As much as 25% of the food produced globally is lost post-harvest due to microbial spoilage^1^. Although a variety of chemical preservatives are extensively used in the food industry to improve the shelf life of food products, many of these have been associated with possible side-effects^2,3^. It is also worrying that while some additives are banned in many countries, others continue to use them. This poses a risk to the exposed populations. It is thus imperative that we move toward natural and safer alternatives to address the specific needs of the area. In this regard, peptide-based antimicrobials are promising due to their enhanced inhibitory activity and greater biocompatibility. Antimicrobial peptides (AMPs) also offer the advantage of preserving food without changing its nutritional and sensory qualities^4,5^. However, in many cases, poor solubility, toxicity, low stability, time-consuming extraction methods, and cost of production are limiting the use of AMPs in food applications. For instance, Nisin is a widely used and commercially exploited antibacterial peptide preservative which has been mainly used in the preservation of dairy and meat products. But there are some confines associated with Nisin that limit its widespread application. Nisin is efficient at acidic pH but loses activity above pH 7. It is also unstable at higher temperatures and shows no activity against Gram-negative bacteria, molds, and yeasts^6^. There is a pressing need to expand the arsenal of peptide molecules tailored for food preservation.

AMPs are generally made up of <50 amino acid residues and show high selectivity and antimicrobial activity against different microorganisms^7^. They have been categorized into several groups based on their length, structure, mode of action, and presence or absence of disulfide bridges^7–9^. Most AMPs are known to inhibit bacterial cells by membrane destabilization and pore formation via specific mechanisms^10,11^. Apart from targeting membranes, several AMPs are reported to target various intracellular biosynthetic processes in bacteria such as the biosynthesis of proteins, DNA, RNA, etc.^12^. The clinical use of most AMPs is limited for reasons such as low in vivo stability, toxicity, or immunogenicity^13–15^. In addition, many AMPs are susceptible to cleavage by trypsin-like proteases, resulting in low efficiency at the site of action. However, they also present the possibility of tailoring the properties and function with relative ease to achieve specific inhibition of a class of microbe. A significant body of research focusing on developing novel antimicrobial peptides is a testament to the interesting possibilities in this area. Some of the groups have isolated and characterized new AMPs from various natural sources and engineered them for better efficacy^16–20^. Several other groups are involved in the de novo design of antimicrobial peptides coupled with structure-function studies to develop safer alternatives to natural AMPs^21–27^. However, peptides having antimicrobial activity, in combination with properties such as low cytotoxicity and hemolytic activity, stability at a wide range of temperature and pH, and low protease susceptibility will be required for successful applications in diverse settings. Most of the efforts, so far, have focused on specific applications in therapeutics and have largely overlooked the needs and possibilities in food preservation and packaging.

In this study, we attempt to design possible alternatives to antimicrobial peptides like Nisin. We ‘rationally’ develop new peptides that simultaneously possess multiple properties like stability at a broad range of pH, thermostability, and anti-trypsin activity. Bowman–Birk inhibitors (BBIs) are a class of serine protease inhibitors that are highly stable^28^. These contain a conserved nine-residue *loop* responsible for protease inhibitory activity. Various BBIs are already being used to contain insect growth^29^ and for therapeutic applications^28,30–35^. Based on our analysis of the structure and activity of this peptide class, our design strategy involves recognizing and incorporating multiple ‘desirable properties’ to obtain robust antibacterial agents with high stability. Preliminary studies suggest that these peptides are safe and could in principle be employed to increase the shelf life of food products. We also attempt to decipher their mode of action. Molecular dynamics (MD) simulation studies on the peptide-membrane complex shed light on the underlying peptide-membrane interactions that result in the antimicrobial action. Overall, this study provides insights into the mechanism of action of such class of peptides and demonstrates how multiple properties can be rationally incorporated to develop superior antibacterial peptides. Most importantly, some of these insights are broadly applicable to the design of antimicrobials for clinical and biomedical applications, in addition to food preservation.

## Results and discussion

### Analysis of natural peptides for the rational design of antibacterial properties

**Fig. 1:**
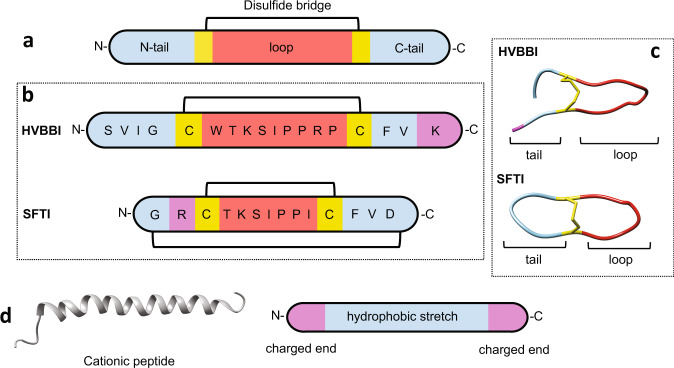
Design of BBI-based antibacterial peptides. **a** A general schematic of Bowman–Birk Inhibitor structure. **b** The peptide exists in a hairpin loop conformation due to formation of disulfide bridge between the loop-flanking cysteines. **c** The structural elements of the peptides as observed in crystal structures. SFTI-PDB ID:1SFI, HVBBI-PDB ID:4U2W. **d** Schematic representation of a cationic peptide structure (Moricin-PDB ID:1KV4). Loop region is indicated by red, cysteines are colored yellow, tail region is colored blue, and cationic residues in the tail region are colored pink.

We started with the analysis of two natural peptides: HVBBI-a β-hairpin BBI found in skin secretions of Chinese bamboo odorous frog, *Huia versabilis*^36^ and SFTI-a bicyclic trypsin inhibitor from sunflower seeds^37^. Common to these peptides is a central trypsin inhibitory loop (hereafter termed simply as ‘*loop’*), flanked by cysteine residues that form a disulfide bridge (Fig. 1a). In addition to the *loop*, there are additional N- and C-terminal *tail* segments attached to the *loop* cysteines (hereafter termed as ‘*tail’*). The two peptides are structurally similar but differ in the nature of amino acids-both within the *loop* and the adjoining *tail* region. In case of HVBBI, the *tail* segments are composed of a cluster of hydrophobic residues and notably, a lysine residue at the C-terminal. Similarly, the terminal segments in SFTI, albeit a little shorter, also possess a couple of hydrophobic residues and an arginine at the N-terminus (Fig. 1b). The peptides exist as a hairpin due to disulfide-bond mediated cyclization of the *loop* region. Several studies have highlighted the importance of disulfide bond for high thermostability of these peptides and their resistance to proteolytic degradation^33^. Due to their net positive charge, these peptides are attracted specifically toward bacterial phospholipid membrane, which is negatively charged due to 23% phosphatidylglycerol (PG) content^38^. Interestingly, HVBBI, but not SFTI, displays a moderate antibacterial activity (Table 1, box A).

**Table 1:** Peptides used in this study. Amino acid sequences, molecular weights, net charges, length, MICs (against *M. luteus*) and Ki values (against bovine trypsin) of the tested peptides.

	SN	Name	Sequence	Charge	Length	MIC (µg/mL) *M. luteus*	*K*_i_ (M)	MW in Daltons
	1	SFTI-cyclic	GRCTKSIPPICFPD	+2	14	–	5.5 ± 0.18 × 10^−8^	1513.6
Box A	2	HVBBI	SVIGCWTKSIPPRPCFVK	+3	18	150	3.7 ± 0.11 × 10^−7^	2016.6
	3	SFTI-loop	CTKSIPPICF	+1	10	–	0.5 ± 0.19 × 10^−6^	1108.3
	4	HVBBI-loop	CWTKSIPPRPCF	+2	12	>150	1.4 ± 0.23 × 10^−6^	1434.6
	5*	HSEP1	SVIGCTKSIPPICFVK	+2	16	75	2.3 ± 0.14 × 10^−7^	1690.2
Box B	6*	HSEP2	SVIFGCTKSIPPICFVGFK	+2	19	6.25	5.8 ± 0.32 × 10^−7^	2041.5
	7*	HSEP3	RSVIFGCTKSIPPICFVGFK	+3	20	1.25	2 ± 0.47 × 10^−7^	2197.6
	8*	FITC-HSEP2	FITC-SVIFGCTKSIPPICFVGFK	+2	19	6.25	–	2543.8
	9*	HSEP2-∆K8	SVIFGCT^I^SIPPICFVGFK	+1	18	>150	NI	1912.6
Box C	10*	HSEP2-K8G	SVIFGCTGSIPPICFVGFK	+1	19	37.5	NI	1969.7
	11*	HSEP2-∆K19	SVIFGCTKSIPPICFVGF^I^	+1	18	25	4.4 ± 0.12 × 10^−7^	1913.3
	12*	HSEP2-ΔHR	A^I^CTKSIPPICG^I^K	+2	12	>150	1 ± 0.09 × 10^−6^	1215.5
	13*	HSEP2-ΔHR- ΔK8	A^I^CT^I^SIPPICG^I^K	+1	11	>150	NI	1087.3
	14*	HSEP3-∆T_L_,C_L_^+^	RSVIFGCYRRFCFVGFK	+4	17	3.125	NI	2083.40
	15*	HSEP3a	RSFIFGCTKSIPPICFVGFK	+3	20	12.5	5.5 ± 0.13 × 10^−8^	2245.50
Box D	16*	HSEP3b	RSVIFGCTKSIPPICFVGTR	+3	20	6.25	0.5 ± 0.38 × 10^−6^	2179.35
	17*	HSEP3c	RSVIFGCTKSKIPPICFVGFK	+4	21	3.125	1.4 ± 0.25 × 10^−6^	2398.35
	18*	HSEP3d	RSWIFCTRYIPPICFVGWR	+3	19	3.125	3.7 ± 0.16 × 10^−7^	2325.40

Underlined residues are the ones that are mutated with respect to the mother peptide.

*Peptides engineered for this study. NI, No Inhibition.

^I^Implies residues deleted from the mother peptide (e.g., those with ΔK).

There are also some important differences. SFTI is a bicyclic peptide formed through a C–N-terminal cyclization, in addition to the disulfide bond (Fig. 1c). The residues comprising the *loop* region within the two peptides are also slightly different. In HVBBI, the *loop* sequence is WTKSIPPRP. This *loop* is responsible for the inhibitory activity of the peptide against extracellular and intracellular serine proteases^16^. The core sequence, TKS is conserved in trypsin inhibitory loops^39^. SFTI has a shorter *loop* sequence TKSIPPI, which also retains the core inhibitory sequence, TKS. Nevertheless, these differences in the *loop* sequences result in different trypsin inhibitory activities (*K*_i_); SFTI shows about 7 times higher *K*_i_ (5.5 × 10^−8^ M) compared to the *K*_i_ of HVBBI (37 × 10^−8^ M) (Table 1). Likewise, we see that the *tail* segments of both peptides show differences in sequence length and residue composition. HVBBI has a slightly higher hydrophobic residue composition compared to SFTI. Although the C-terminal lysine residue observed in HVBBI is missing in SFTI, the function associated with it, i.e., specific binding to bacterial membranes, could in principle be taken over by the arginine near the N-terminal end of SFTI. The *tail* segment—consisting of cationic residues together with hydrophobic segments—are also common in natural cationic antibacterial peptides^17^. Previous studies on cationic peptides have attributed their antibacterial, membrane destabilizing activity to the presence of basic (cationic) residues and a hydrophobic region (Fig. 1d)^17^. These peptides are thought to assume an amphiphilic structure, wherein the positively charged ends interact with the negatively charged polar head-groups, while the hydrophobic core interacts with the lipid acyl chains. From this, it follows that a greater number of cationic residues and/or hydrophobic residues would enable a stronger interaction between peptides and the phospholipid bilayer. Notably, the net positive charge on HVBBI is also higher compared to the net charge on SFTI (Table 1, box A).

We set out to understand how the aforementioned differences in HVBBI and SFTI affect their antibacterial activities. It is important to mention here that antimicrobial peptides are known to demonstrate significantly different antimicrobial activities or minimum inhibitory concentrations (MICs) against different bacterial species, even when they are closely related. Therefore, we use Gram-positive bacterium, *Micrococcus luteus*—one of the widespread food spoilage bacteria—as a simple model organism to compare the efficiency (MIC values) of the various peptides designed in this study. HVBBI shows an MIC value of 150 µg/mL against *M. luteus*, whereas SFTI shows no detectable inhibitory activity within the assayed concentration range (Table 1, box A). Furthermore, removing the *tail* segment from HVBBI peptide drastically compromises the antibacterial activity (HVBBI-*loop*, Table 1, box A). As expected for SFTI-*loop*, no antibacterial activity was observed upon removing the *tail*. Interestingly, for both SFTI and HVBBI, the removal of *tail* segments affected the trypsin inhibitory constant by 2 orders of magnitude (compare *K*_i_ values in Table 1, box A). This suggests that apart from its role in antibacterial activity, the *tail* segment also influences the anti-trypsin activity of the peptide.

Rational design of peptides with better antimicrobial properties requires an understanding of how the sequence and structure of the peptides may be related to their bioactivity. From the above discussion and previous studies on cationic antimicrobial peptides^40–43^, we reasoned that increasing the hydrophobic core and the positive charge would result in better antibacterial activity. Interestingly, besides small peptides like HVBBI and SFTI, BBIs are usually found as 8, 16, and 24 kDa proteins with a minimal repeating 4 kDa unit^44^. In the larger BBIs, the *loop* is flanked by two beta strands on either side, which are linked by one or more disulfide bridges that further enhance the stability. In the case of short peptides, the beta strands (*tail* segment) can be designed with higher hydrophobic composition and/or greater cationic charge for improved antibacterial activity through side-chain mutations or insertion of additional residues.

**Fig. 2:**
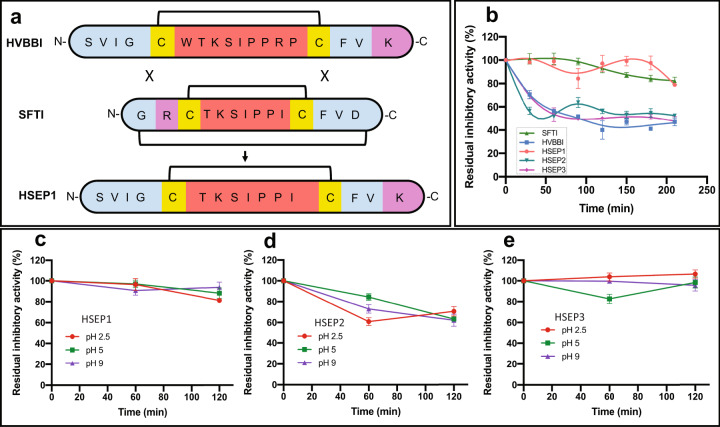
Thermostability and pH stability of the designed peptides measured through trypsin inhibition assays. **a** A chimeric peptide, HSEP1 derived by interchanging the extra-TIL segment of SFTI with that of HVBBI. Loop region is indicated by red, cysteines are colored yellow, tail region is colored blue, and cationic residues in the tail region are colored pink. **b** Thermostability profiles of the peptides. **c**, **d**, **e** pH-stability profiles for HSEP1, HSEP2, and HSEP3, respectively. Data represent mean ± s.e.m. of two biological replicates.

To test our hypothesis, we first designed a chimeric peptide, HSEP1—derived from HVBBI and SFTI, wherein the *tail* of SFTI was interchanged with that from HVBBI (Fig. 2a). SFTI-*loop* has about three times higher binding affinity for trypsin compared to HVBBI-*loop* (Table 1, box A). At the same time, HVBBI has a higher antibacterial activity, which may be attributed to its longer *tail* region. We reasoned that replacing the SFTI-*tail* with the HVBBI-*tail*, in principle, yields a peptide with a better overall antibacterial activity. Table 1 (box B) shows that the trypsin inhibitory constant obtained for the new peptide—HSEP1 was 23 × 10^−8^ M, which is similar to the value obtained for HVBBI. However, the antimicrobial activity of the chimeric peptide HSEP1 (MIC: 75 µg/mL) also showed improvement over that of HVBBI (150 µg/mL).

It is interesting to compare HSEP1 and HVBBI sequences in the context of their measured antibacterial activities. The two peptides differ only in the loop region. If one assumes that the antibacterial activity of the peptide was entirely dependent on the *tail* segments, the MIC values for both peptides should be similar. This however is not the case, as HSEP1 shows enhanced antibacterial effect compared to HVBBI (Table 1). This is despite the lower basicity (charge +2) of HSEP1 compared to that of HVBBI (+3); this leads to an interesting possibility of the *loop* region influencing the overall antibacterial activity of the peptide. It appears that the *loop* and the *tail* segments both influence the antibacterial as well as the anti-trypsin activities of the peptides; the exact factors governing this influence need to be understood. In summary, supplementing the SFTI-*loop* with HVBBI-*tail* resulted in a peptide, HSEP1, with better antimicrobial efficacy against *M. luteus*.

### Improving upon sequence designs for better antimicrobial efficacy

We next sought to improve the HSEP1 activity by varying physiochemical properties such as hydrophobicity and net charge. First, we increased the hydrophobicity of HSEP1. It is understood that the hydrophobic residues aid in the interaction of the peptides with the acyl chain of the lipids. Moreover, mutual aggregation of peptides through these hydrophobic regions has been suggested to aid the membrane destabilization process^45^. Of all hydrophobic residues, aromatic phenylalanine residue is particularly interesting as it has been reported to enhance mutual aggregation of the peptides on membrane surfaces^16^. Therefore, we introduced additional phenylalanine residues in both the N-*tail* (note, in HSPE2 that a Phe is inserted between Ile3 and Gly4 of HSEP1) and C-*tail* regions (note, in HSEP2 a Gly-Phe inserted between Val15 and Lys16 of HSEP1) to boost the hydrophobic character of the HSEP1 peptide (Table 1) (HSEP1: SVIGCTKSIPPICFVK and HSEP2: SVI**F**GCTKSIPPICFV**GF**K). Notice that in the C-*tail* region, we introduced an extra glycine residue as well. As bulky residues like phenylalanine introduce conformational restrictions that may hinder efficient peptide aggregation, an extra glycine was added to offset some of the backbone rigidity due to the two additional phenylalanine residues. Indeed, the redesigned peptide, HSEP2, exhibits significant antibacterial activity (6.25 µg/mL). Notably, the incorporation of additional phenylalanine residues affects the trypsin inhibitory constant of HSEP2 (54 × 10^−8^ M) only marginally compared to that of HSEP1 (23 × 10^−8^ M) (Table 1, box B).

We next sought to improve HSEP2 further by enhancing the net positive charge. We tested the variant HSEP3 wherein we introduced a basic residue (arginine) at the N-terminal end of HSEP2 to enhance its cationic character. This peptide exhibited the best antibacterial activity (1.25 µg/mL). Comparison of the sequence and activities of the three designed peptides (see HSEP1, HSEP2, and HSEP3 in Table 1, box B) suggests that the *tail* segment—common to all three peptides and including the hydrophobic core and terminal basic residues—is critical for the antibacterial activity of the peptide.

### Hyperstability of HSEP3

The presence of a disulfide bridge imparts thermostability to the peptides—a desirable property for food preservation application. We determined the stability of the designed peptides at high temperature. HSPE3 exhibited ~30% decrease in trypsin inhibitory activity within the first 30 min of incubation at 95 °C (Fig. 2b) and retained 50% of its activity after heating at 95 °C for 200 min. We also tested the effect of temperature on the antibacterial activity of HSEP3. HSEP3 did not show any detectable reduction in the MIC upon dry or moist heat treatment (Supplementary Table 2). For testing pH stability, the peptides were incubated at three different pH [at pH 2.5, pH 5, and pH 9] (Fig. 2c, d and e). HSEP3 retained more than 80% of its trypsin inhibitory activity at all three pH values (Fig. 2e).

### Characterization of designed peptides and their mode of action

**Fig. 3:**
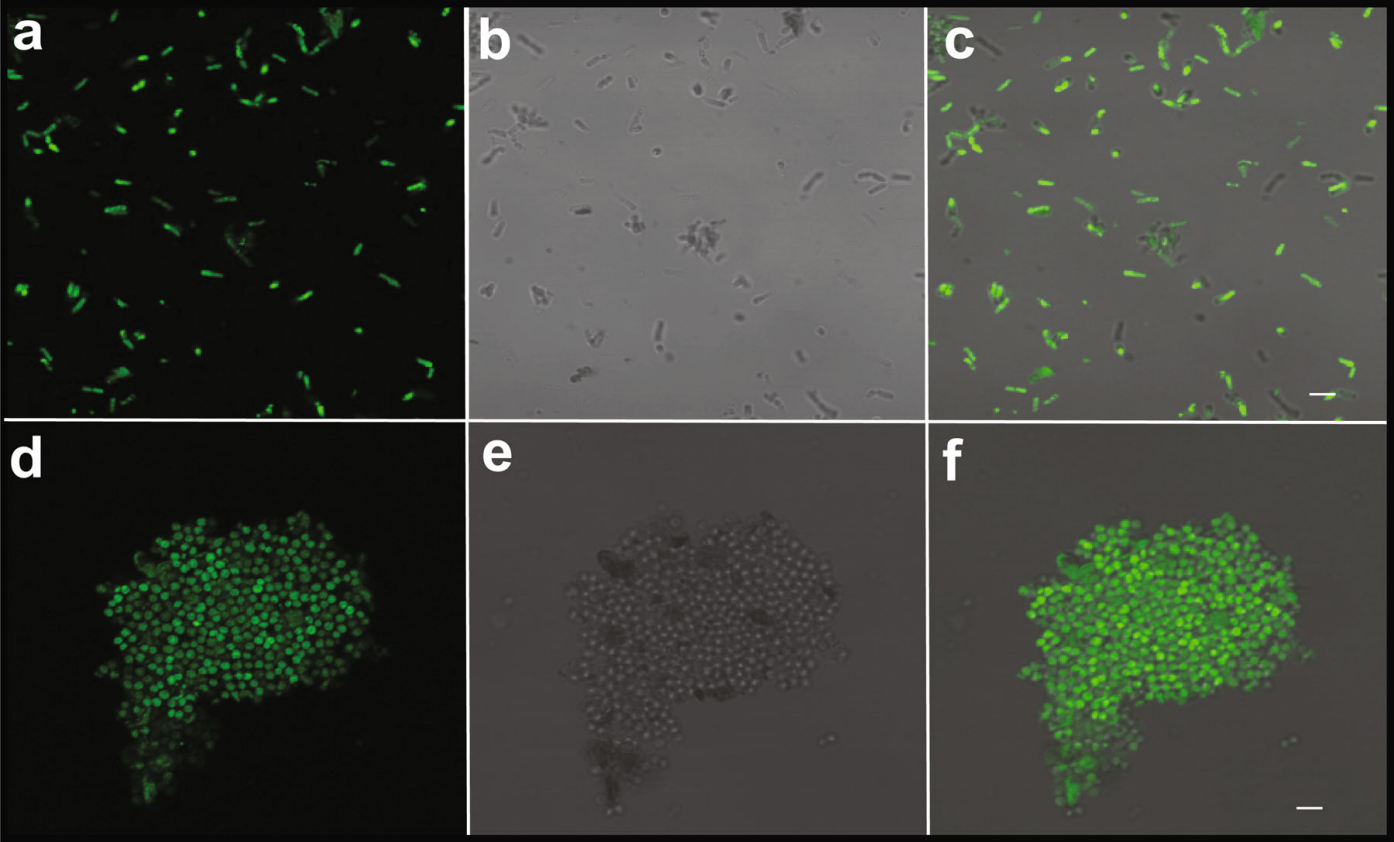
Confocal images of cells incubated with FITC-HSEP2 at 2X MIC. Confocal images of *B. cereus*: **a** Fluorescence image, **b** DIC image, and **c** merged image. Confocal images of *M. luteus*: **d** Fluorescence image, **e** DIC image, and **f** merged image. For figures **a**–**c**, scale bar denotes 5.2 μm and for figures **d**–**f**, scale bar denotes 6.75 μm.

**Fig. 4:**
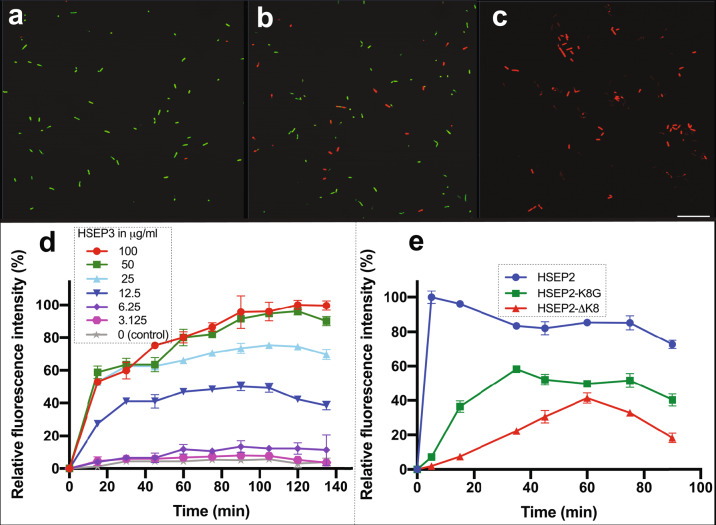
Membrane permeabilization in *B. cereus* treated with HSEP3, HSEP2, and HSEP2-mutant peptides. Upper panel: **a** Control, no peptide; **b** HSEP3 treated at 0.5X MIC concentration, and **c** HSEP3 treated at 1X MIC concentration. All three samples treated with Syto-9 (in green, indicating live cells) and propidium iodide (in red, indicating dead cells). **d** Permeabilization of the cytoplasmic membrane of *B. cereus* as a function of HSEP3 peptide concentrations (0–100 μg/mL), calculated as percent of propidium iodide fluorescence. **e** Permeabilization of the cytoplasmic membrane of *B. cereus* by HSEP2, HSEP2-K8G, and HSEP2-∆K8 (2X MIC), indicated by percent of propidium iodide fluorescence. Data represent mean ± s.e.m. of three biological replicates. Scale bar denotes 25.40 μm.

The designed peptides fall under the class of cationic peptides that cause membrane destabilization. We performed localization studies on one of the peptides, HSEP2, using confocal laser-scanning microscopy. We first designed FITC tagged HSEP2 (FITC-HSEP2) and checked that FITC-tag had no effect on the antibacterial activity of the peptide (Table 1). For microscopy studies, we used *B. cereus* in addition to *M. luteus*. We verified that HSEP2 and HSEP3 show significant MIC values against *B. cereus* (HSEP2: 75 µg/mL, HSEP3: 12.25 µg/mL; Table 2). Confocal images of cells depict the bacterial cells (*B. cereus* and *M. luteus*) in fluorescent green color, indicating that the peptide indeed targets the bacterial cell membrane (Fig. 3). Moreover, live-dead staining shows that the peptides increase membrane permeability. In this assay, a cell with a damaged membrane takes up PI and fluoresces red; the live cells bind SYTO 9 and fluoresce green. HSEP3-treated cells appeared red indicating membrane permeability and cell death (Fig. 4a, b, c and Supplementary Figure 1).

**Table 2:** MICs of the peptides against tested bacteria.

S No.	Microorganisms	HSEP2	HSEP3	HSEP2-∆K19	HSEP3-∆T_L_, C_L_^+^	HSEP3a	HSEP3b	HSEP3c	HSEP3d
Gram-positive									
1	*Listeria monocytogenes*	>150	50	>150	>150	>150	100	50	>150
2	*Bacillus cereus*	75	12.5	>150	>150	>150	50	100	100
3	*Staphylococcus aureus*	>150	150	>150	>150	>150	>150	150	>150
4	*Micrococcus luteus*	6.25	1.25	25	3.125	12.5	6.25	3.125	3.125
Gram-negative									
5	*Escherichia coli*	150	150	>150	>150	>150	>150	100	>150
6	*Pectobacterium carotovorum*	150	50	>150	>150	>150	50	50	>150
7	*Salmonella typhimurium*	>150	85	>150	>150	>150	>150	>150	>150

Minimum inhibitory concentrations (MICs) were determined as the lowest concentration of the peptide that inhibited bacteria growth. MICs are expressed in µg/mL.

We further monitored the membrane destabilization event over time through a simple propidium iodide (PI) uptake assay using *B. cereus* cells. Herein, we measured the total fluorescence emitted by dead cells over a time-course of 2 h. HSEP3-treated *B. cereus* cells become permeable to PI with increased fluorescence within the first 5 min (Fig. 4d), with signal saturation in 45 minutes. Moreover, the PI fluorescence also showed a dose dependency. At a concentration of 6.25 and 3.125 μg/mL of HSEP3, the fluorescence intensity was at the basal level. At 12.5 μg/mL, fluorescence increased significantly; this correlates with the MIC for HSEP3 peptide against *B. cereus*. A further increase in concentration resulted in increased PI uptake; however, after 50 μg/mL concentration, the PI fluorescence reached a plateau.

**Fig. 5:**
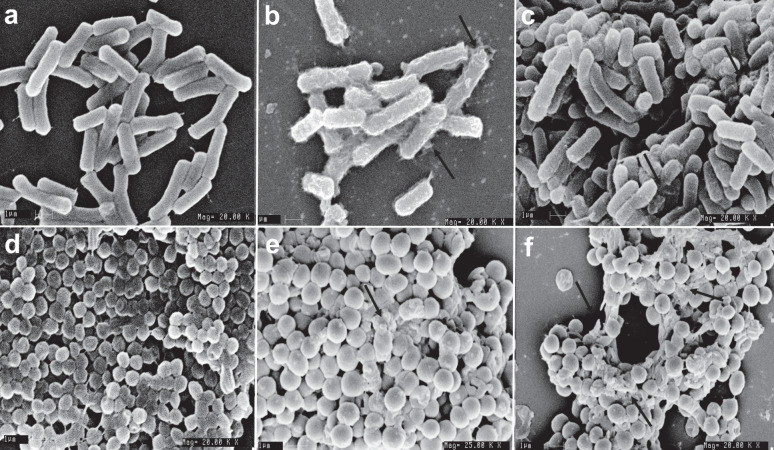
Scanning electron micrographs (SEM) of *B. cereus* and *M. luteus* treated with the peptides. Upper panels—SEM micrographs of *B. cereus*: **a** Control, no peptide; **b** HSEP2-treated; **c** HSEP3-treated. Lower panels—SEM micrographs of *M. luteus*: **d** Control, no peptide; **e** HSEP2-treated (at ×25 magnification, while all others are at ×20); **f** HSEP3-treated. Note that for all images the scale bar denotes 1 μm.

**Fig. 6:**
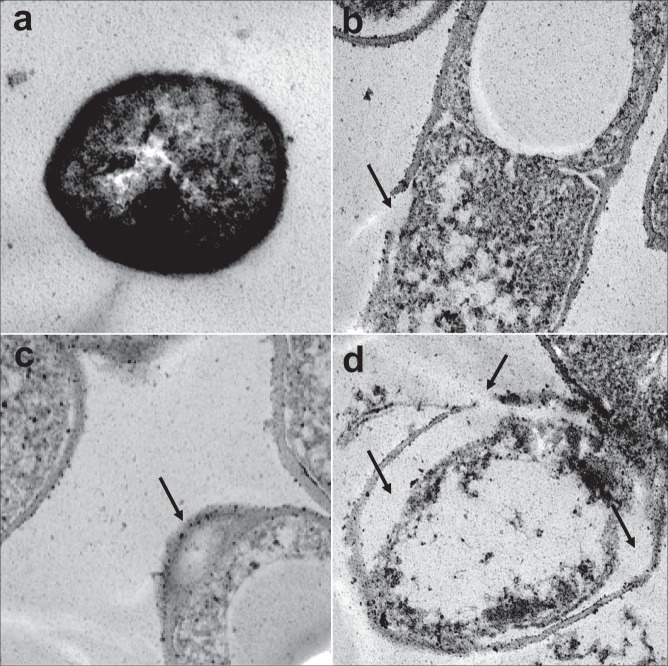
Transmission electron micrographs (TEM) of *B. cereus* treated with HSEP3. TEM micrographs of *B. cereus*: **a** represents control (without peptide); **b**, **c**, and **d** represent different micrographs for HSEP3-treated cells (different fields from the same sample).

Finally, we observed changes in bacterial cell membrane morphology on treatment with peptides, as evident from scanning electron microscopy (SEM) imaging. Membrane surface of cells treated with peptides had a corrugated appearance and intracellular content leakage was observed (Fig. 5). In contrast, the negative control cells had a smooth appearance and had no signs of cell damage or intracellular leakage. Although the SEM images revealed membrane corrugations and cell leakage, the resolution was insufficient to discern membrane damage and pore formation on the cells. Transmission electron microscopy (TEM) was further carried out to obtain high-resolution micrographs of bacterial cells treated with HSEP3. Untreated cells showed normal cell shape with an undamaged inner and outer membrane ultrastructure. Cells were characterized by an increased electron density for the cytosol. The intracellular region exhibited a highly heterogeneous electron density, and no apparent damage or cytoplasmic leakage was observed in case of the untreated cells. In contrast, TEM micrographs of HSEP3-treated bacterial cells show distinctive cytoplasm-devoid zones, homogenous electron density, and loss of membrane integrity with clearly visible pores on the outer membrane (Fig. 6).

### Simulation studies on lipid-peptide-water ternary system

To gain atomic-level picture of the peptide interaction with bacterial phospholipid membrane, we performed all-atom MD simulations on a system consisting of HSEP3 peptides in association with POPE-POPG bilayer. POPE-POPG bilayer was used previously as a model for a negatively charged bacterial membrane in studies on cationic peptides^46,47^. Prior to peptide-membrane simulations, we performed simulations on POPE-POPG bilayer of different sizes—with 200, 400, and 800 lipids (see “Methods”)—to ensure that the bilayer properties such as bilayer thickness (*D*_*P-P*_) and area-per-lipid (*A*_*L*_) converged to stable and expected values (Supplementary Table 1). The calculated ensemble-averaged values of *A*_*L*_ and *D*_*P-P*_, and *K*_*A*_ (isothermal area compressibility modulus) from the simulations are tabulated in Supplementary Table 1 and are close to previous reported values^48–51^.

Since we obtained similar equilibrium properties for all three systems sizes, we chose the median 400-lipid system, for peptide-membrane simulations. We simulated a membrane-peptide-water system for 1.5 µs wherein the peptides were already bound to the upper leaflet of the membrane (Supplementary Figure 2). The ensemble-average values of *A*_*L*_ obtained for the peptide-bound membrane were 60.87 ± 0.75 Å^2^, and the calculated *K*_*A*_ of about 230 mN/m. The average bilayer thickness (*D*_*P-P*_) was calculated to be 3.83 ± 0.18 nm. Comparison of these values with those calculated from a 300 ns control simulation of peptide-free membrane indicates that binding of HSEP3 to the membrane causes a decrease in the average membrane thickness by ~3 Å (*D*_*P-P*_ = 4.12 ± 0.03 nm for peptide-free membrane). However, simulations did not indicate a significant change in *A*_*L*_ values calculated for the peptide-free (*A*_*L*_ = 59.84 ± 0.64 Å^2^) and peptide-bound membrane (*A*_*L*_ = 60.87 ± 0.75 Å^2^). Previous studies have reported significant changes in the area-per-lipid values in case of membranes bound to peptide^52^. However, this modulatory effect of peptide binding on the membrane property varies with the peptide concentration used and is therefore expected to increase in simulations with higher peptide concentrations.

**Fig. 7:**
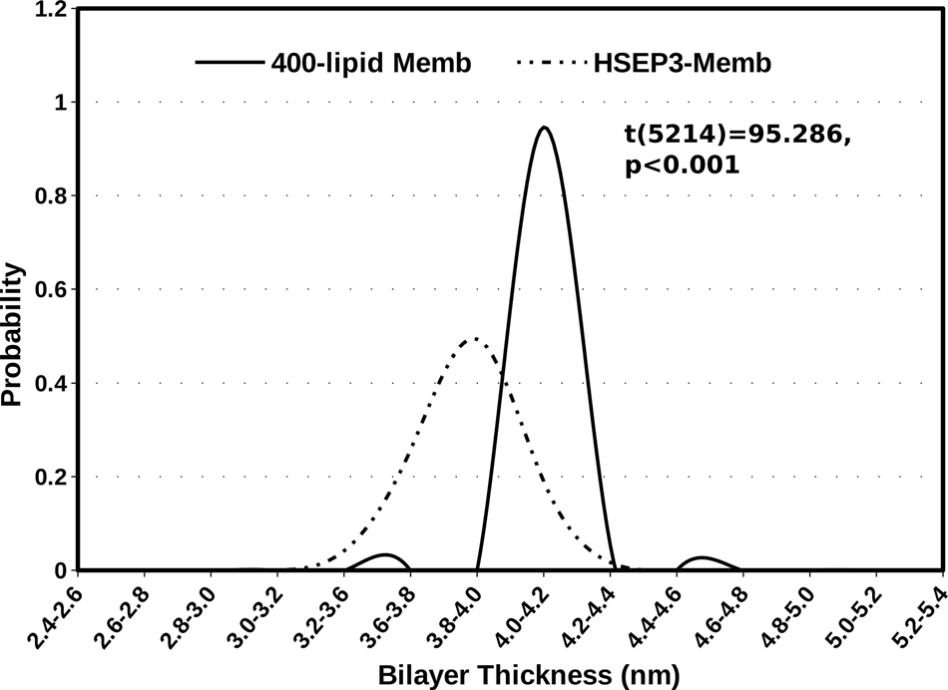
Distribution of bilayer thickness values (*D*_*P-P*_) for trajectory snapshots for membrane and HSEP3-bound membrane simulations. A two-sample *t*-test assuming unequal variances shows that the mean membrane thickness values are significantly different (two-sample t(df=5213) = 95.286, *p* < 0.001).

**Fig. 8:**
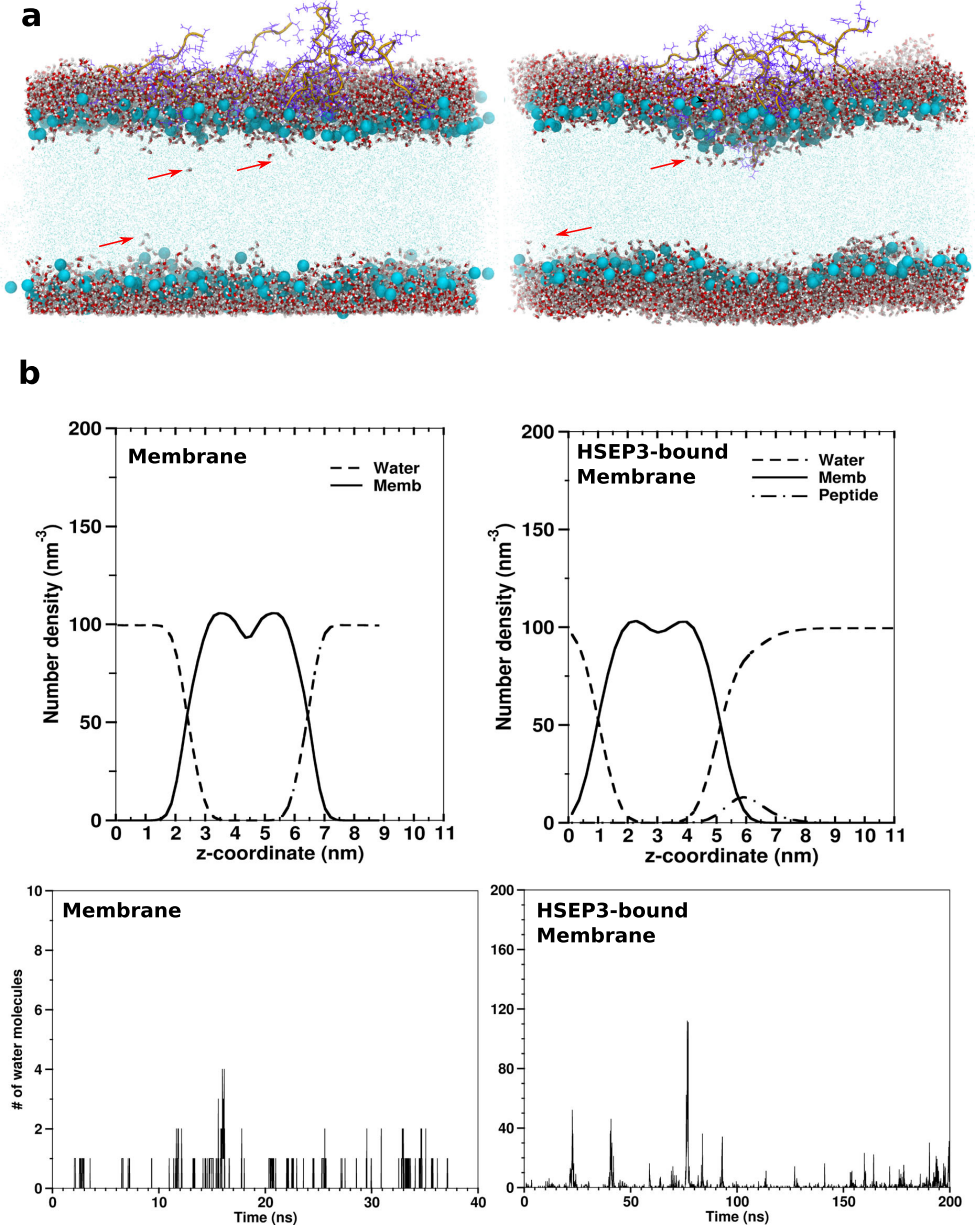
Water permeability of HSEP3-bound membranes. **a** Trajectory snapshots showing water insertion defects in HSEP3-bound membranes. **b** Upper panels: Partial number density profile for peptide-free and HSEP3-bound membrane simulations. Lower panels: Number of water molecules within hydrophobic region (the central 2.0 nm) of the bilayer per trajectory frame.

Visual analysis of the simulation trajectory revealed that HSEP3 peptides undergo aggregation on the upper leaflet surface of the bilayer, which seems to disrupt lipid packing and cause membrane-surface deformation. The distribution for the average bilayer thickness shows that the peptide-bound membrane has a lower average bilayer thickness with a higher variance (0.024 nm) compared to peptide-free membrane system (0.0021 nm) (Fig. 7). Moreover, we observed a drastic increase in water permeation and water insertion defects due to membrane deformations in case of the peptide-bound membrane, indicating higher water permeability (Fig. 8). Similar observations have been noted previously for other membrane destabilizing molecules such as Maculatin^53^. We also calculated the lipid acyl-chain order parameter, *S*_*CD*_ for the peptide-bound and free membrane. Order parameters measure the chain ordering and higher chain ordering is correlated with greater lipid packing and bilayer thickness. The *S*_*CD*_ plots (Supplementary Figure 3) also indicate differences in the order parameters values for the acyl-chain carbon atoms of the peptide-bound membrane, compared with the corresponding values for the peptide-free membrane. Based on these results, it can be concluded that interaction of the membrane with the peptide disrupts proper lipid packing and subsequently leads to a decrease in membrane thickness. The aforesaid effect on lipid acyl-chain ordering leads to formation of transient defects in the membrane structure that become accessible to water molecules.

**Fig. 9:**
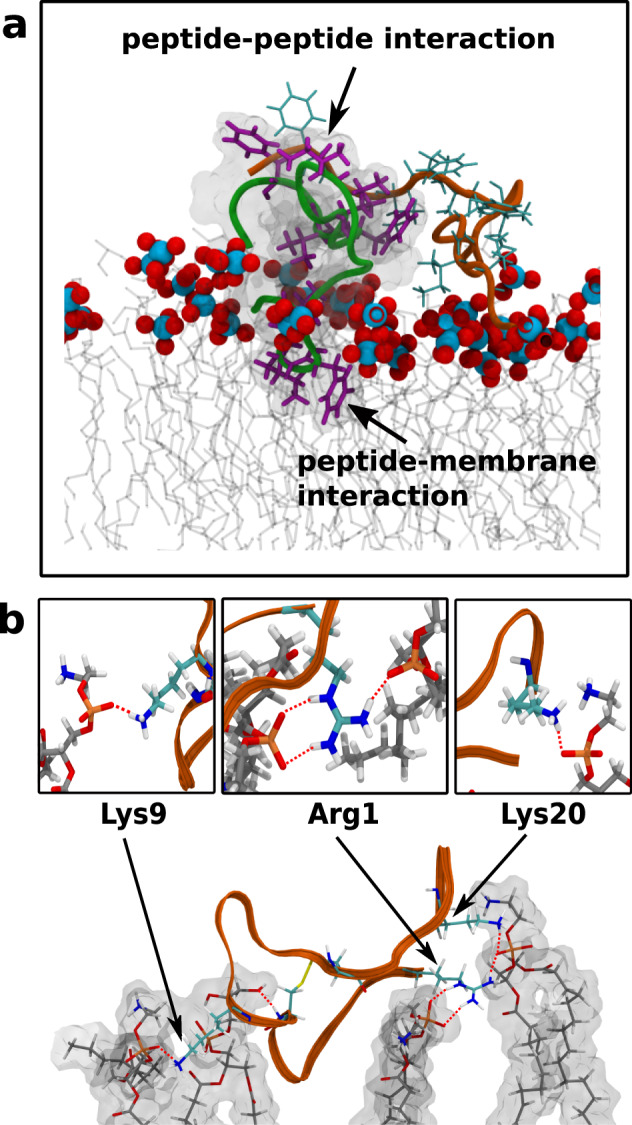
Observed peptide-membrane interactions. **a** Hydrophobic residues involved in peptide-peptide and peptide-membrane interactions. Two peptides (in green and orange ribbon) and their respective hydrophobic residues are depicted (in magenta and cyan sticks). Blue-red balls denote lipid phosphate groups, while the acyl chains are depicted in gray lines. **b** Polar interactions between HSEP3 peptide (ribbon in orange) and the membrane lipids (sticks) as observed in the final snapshot of simulation. The h-bond interactions (red line, dotted) are chiefly mediated by the cationic residues—lysine and arginine.

The designed HSEP3 peptide belongs to the class of cationic peptides as they possess a net positive charge. Higher positive charge enhances the binding of the peptides to the membrane. Analysis of the trajectory reveals that the peptides form on an average of 3–4 hydrogen bonds per peptide through lysine and arginine residues—amounting to roughly 6–8 kcal/mol of binding energy per peptide. The typical interactions formed between the peptide and membrane lipids are depicted in Fig. 9. This apart, the peptides comprise a number of hydrophobic residues; we have already demonstrated that the hydrophobic residues are essential for effective antimicrobial activity.

Our simulations show that HSEP3 exerts its membrane destabilizing activity by aggregating on membrane surface, leading to bilayer thinning and increased water permeation into the bilayer. For the effective number of peptides used in the simulations, we did not observe any membrane-insertion events in case of HSEP3 within the timescale of these simulations. There is experimental evidence that the exact mechanism of membrane disruption is dependent on lipid:peptide ratio and membrane composition^54^. Recent work by Tieleman and coworkers indicates the absence of pore-forming membrane-insertion events in long simulations using the CHARMM force-field^55^. Nonetheless, the simulation results support the ‘carpet model’ as a plausible membrane disruption mechanism employed by HSEP3. According to this model, the peptides bind and accumulate on the membrane surface like a ‘carpet’ and at high concentrations, the peptides disrupt the bilayer by causing membrane defects.

### Investigating the role of the ‘*loop*’ in antibacterial function

The peptides have high inhibitory activity against trypsin owing to the *loop* region. Our observations in previous sections suggest that the peptides cause membrane disruptions leading to cell death, but the role of the *loop* region in the overall antimicrobial effect is unclear. Fluorescence experiments show that the peptides are internalized by the cells, which further indicates they may inhibit intracellular serine proteases. To evaluate this, we measured trypsin activity of bacterial cell lysate with and without peptide treatment. While the latter showed significant trypsin activity, peptide-treated lysates exhibited dose-dependent attenuation of intracellular trypsin activity (Supplementary Figure 4). This raises an interesting possibility; the peptides, in addition to binding and localizing at the membrane, enter the cytoplasm and inhibit intracellular trypsin. However, it is still not clear whether the trypsin inhibition per se plays any role in bacterial inhibition. Therefore, we next probed the link between trypsin inhibition exerted by the *loop* and the antibacterial efficacy of the peptide.

To this end, we sought to abolish the anti-trypsin activity through a lysine mutation at the TKS motif within the *loop* and examine the antibacterial activity of the mutant peptide. For our experiments, we created two mutants of HSEP2 (not HSPE3, as HSEP2 was the best peptide design at the time these experiments were conceived); one with a lysine deletion (HSEP2-ΔK8) and the other with a lysine to glycine mutation (HSEP2-K8G). As anticipated, both peptides failed to show any trypsin inhibition. However, in terms of antibacterial property, the two peptides behave differently. HSEP2-ΔK8 did not show any antibacterial activity, while HSEP2-K8G retained significant antibacterial activity, although less effective than HSEP2 (Table 1, box C). We also compared the fluorescence (PI uptake) for bacterial cells treated with peptides—HSEP2 and its mutants (HSEP2-ΔK8 and HSEP2-K8G). Results obtained agree with the antibacterial activity of these peptides; HSEP2-treated cells gave the highest fluorescence intensity indicating membrane destabilization and cell death. HSEP2-ΔK8 and HSEP2-K8G treated cells gave minimal fluorescence intensity, albeit higher in case of HSEP2-K8G, indicating marginal cell death (Fig. 4e and Supplementary Figure 5). These effects may be rationalized as follows. Both the deletion (HSEP2-ΔK8) and point mutant (HSEP2-K8G) variants have decreased net charge (by +1). Indeed, this is expected to affect the antibacterial efficacy of both peptide variants. More importantly, the deletion of lysine leads to a significantly perturbed *loop* structure due to a shortened length, which likely abrogates peptide-trypsin binding. In comparison, a significant antimicrobial activity seen for HSEP2-K8G could be because the mutation of lysine to glycine (in HSEP2-K8G) does not alter the loop structure significantly.

To delineate whether the TKS lysine contributes to antibacterial activity via charge effects, we also tested a HSEP2 peptide variant, HSEP2-ΔK19 wherein the C-terminal lysine was deleted without altering K8 (Table 1, box C). This peptide variant retains the overall net charge of +1, as HSEP2-K8G. HSEP2-ΔK19 demonstrated similar antibacterial activity (25 μg/mL) as HSEP2-K8G (37.5 μg/mL). While it is difficult to precisely pinpoint the roles, it appears that both K8 and the terminal lysine K19 contribute to antibacterial activity through charge effects that directly aid peptide binding to bacterial membrane. Indeed, HSEP3 with an additional arginine residue showed a better MIC value (1.25 μg/mL). We further redesigned the superior HSEP3 peptide such that it loses anti-trypsin activity, without altering the net charge and hydrophobicity. Particularly, we replaced the *loop* sequence (TSKIPPI) with a non-specific sequence (YRRF) that is expected to abrogate the anti-trypsin activity and at the same time compensate for the charge and hydrophobicity. We tested the new peptide, HSEP3-∆T_L_,C_L_^+^ for its anti-trypsin and antibacterial activity [Note: T_L_ stands for Trypsin Loop and C_L_^+^ stands for the compensated charge]. As expected, it did not show any anti-trypsin activity, but still possessed good antibacterial activity (Table 1). Interestingly, the MIC value for HSEP3-∆T_L_,C_L_^+^ (3.125 μg/mL) was better than that of HSEP2-ΔK8, HSEP2-K8G, and HSEP2- ΔK19 peptides. The results show that the residues introduced in place of the *loop* sequence can, to an extent, substitute for its loss via charge and hydrophobicity effects. However, trypsin inhibition appears important, given the complete loss in antibacterial activity by HSEP2-ΔK8. Overall, it appears that the *loop* contributes to antibacterial activity through physiochemical (charge and hydrophobicity) effects causing membrane disruption, and intracellular trypsin inhibitory effects, the former being a dominant factor. The interplay of the two factors appear to govern the overall activity of the peptides.

We investigated this further by rephrasing the problem statement as follows. Of the total amount of peptide required for antibacterial inhibition, what fraction is sufficient for membrane destabilization, so that the remaining fraction can be assumed to be available for intracellular trypsin inhibition? Toward this, we made the following assumption. Out of the total amount of peptide required for inhibition, say *x* (equivalent to the MIC value), only a fraction of the peptides (*a*/*x*) is sufficient for membrane destabilization effects, while the rest of the peptide fraction (*y* = 1−*a*/*x*) is free to diffuse into the cytoplasm through the damaged membrane and inhibit intracellular trypsin. Under this assumption, we hypothesized that if the peptide fraction, *y*, is replaced by a different peptide that possesses only trypsin inhibitory activity, i.e., HSEP2-ΔHR (denoting that the Hydrophobic Region is deleted) then the effective antibacterial efficacy of the resulting cocktail of peptides should be the same as that observed for the total peptide (*x*). To test this hypothesis, we prepared cocktails of HSEP3 and HSEP2-ΔHR in ratios [HSEP3:HSEP2-ΔHR] of 0:4, 1:3, 2:2, 3:1, and 4:0 (for peptide sequences and properties, see Tables 1 and 3). Note that HSEP2-ΔHR does not possess any detectable antibacterial activity but retains reasonably high trypsin inhibitory activity. Here, 0:4 and 4:0 ratio cocktails represent pure HSEP2-ΔHR and HSEP3, respectively; and 1:3, 2:2, and 3:1 are the test cocktails in varying ratios. We tested the antibacterial activity of these cocktails against both *B. cereus* and *M. luteus*. The results tabulated in Table 3 show that out of the three tested cocktails, the one with ratio 3:1 achieves the same activity as observed with pure HSEP3 preparations (i.e., 4:0). The observed trend was the same for both bacterial species. From this, it is evident that about 75% of the total peptide is sufficient for membrane destabilization, while the rest 25% is involved in trypsin inhibition. Further, we tested another set of cocktails prepared from HSEP3 and HSEP2-ΔHR-ΔK8, which represents the inactive form of HSEP2-ΔHR without any trypsin inhibitory activity (as the lysine K8 is mutated here). As expected, the results demonstrate an increase in MIC values against both *B. cereus* and *M. luteus*, at this 3:1 ratio.

**Table 3:** MICs for the peptide combinations against *B. cereus and M. luteus.* MICs were determined as the lowest dilution of the peptide cocktail that inhibited bacterial growth.

S No.	Peptide cocktail ratio	HSEP3:HSEP2-∆HR MIC (µg/mL) *B. cereus*	HSEP3:HSEP2-∆HR MIC (µg/mL) *M. luteus*	HSEP3:HSEP2-∆HR,∆K8 MIC (µg/mL) *B. cereus*	HSEP3:HSEP2-∆HR,∆K8 MIC (µg/mL) *M. luteus*
1	(0:4)	–	–	–	–
2	(1:3)	50	5	50	5
3	(2:2)	25	2.5	25	2.5
4	(3:1)	12.5	1.25	25	2.5
5	(4:0)	12.5	1.25	12.5	1.25

These results pose important implications to the design of synergistic cocktails with antibacterial properties, wherein the surplus fraction of the peptide (*y* = 1−*a*/*x*) may be replaced by another antibacterial molecule with a different mode of action, for instance, the inhibition of another key bacterial function. Previous studies on synergistic action of drugs have concluded that the combination of two drugs often leads to better activity for the combined preparation as compared to the individual activities of parent molecules^56,57^. Further studies are required that test cocktails of HSEP3 in combination with other antimicrobials; such studies may facilitate the identification of effective combinations that possess high antibacterial activity.

### Design of HSEP3 variants: modulating peptide properties and efficacy

We know that HSEP3-membrane interactions are mediated chiefly via polar bonds, weak van-der-Waals forces, and stronger hydrophobic interactions. Thus, modulating the properties of the peptide, such as number of cationic and polar amino acids and hydrophobicity, may in turn alter the antibacterial activity of the peptide; particularly, the introduction of arginine residue and/or hydrophobic residues such as phenylalanine and isoleucine. We created a small set of four HSEP3 variants, which were mutated to have (a) an increased hydrophobicity [HSEP3a (*HSEP3-Val3Phe*)], (b) higher polar residues [HSEP3b (*HSEP3-Phe19Thr*)], and (c) increased cationic charge [HSEP3c: Lys inserted between Ser10 and Ile11 of HSPE3]. We also designed a peptide variant, HSEP3d with mutations that introduced arginine (for greater H-bonding propensity) in place of lysine (HSEP3-Lys9Arg, HSEP3-Lys20Arg), tryptophan (for more hydrophobicity) in lieu of phenylalanine and valine (HSEP3-Val3Trp, HSEP2-Phe19Trp), and a bulkier tyrosine (for additional stacking interactions) in place of serine (HSEP3-Ser10Tyr). Out of these set of peptides, HSEP3c and HSEP3d demonstrated the best activity against *M. luteus* (3.125 µg/mL), whereas HSEP3b was reasonably effective (6.25 µg/mL). All peptides showed reasonable trypsin inhibitory activity. We also tested these peptides against a broad range of bacteria and observed that some of these peptide variants (HSEP3b and HSEP3c) also show efficacy against other microorganisms such as *Listeria* and *Pectobacterium* (Table 2).

### Cytotoxicity and hemolytic assays

**Fig. 10:**
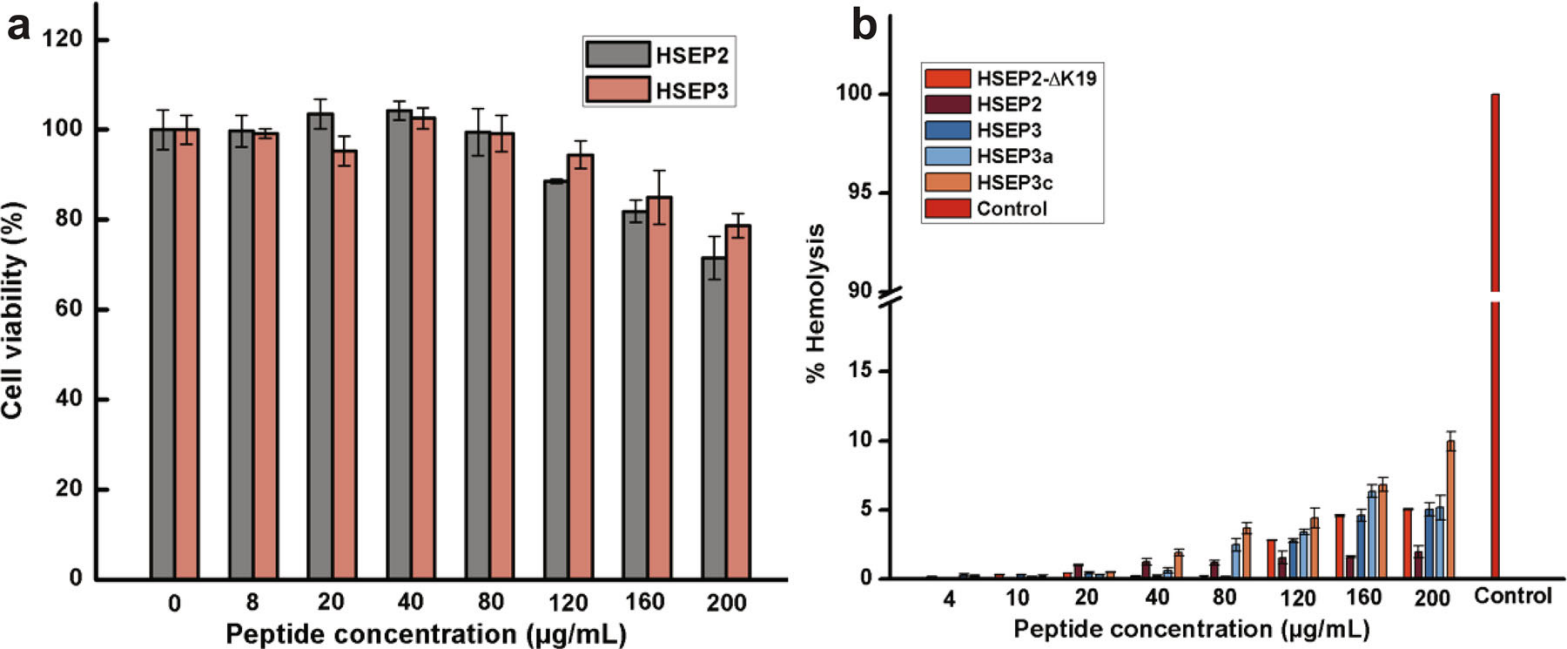
Cytotoxicity and hemolytic activity of the peptides. **a** Cytotoxicity of the peptides. cell viability of ARPE-19 treated with HSEP3 and HSEP2. **b** Percent hemolysis caused by the peptides after incubated with 4% RBCs in PBS. RBCs incubated with 0.1% Triton X-100 was the control showing 100% lysis. Data represent mean ± s.e.m. of three biological replicates.

We tested the safety of selected peptides by cytotoxicity assay on two different human cells lines, human retinal pigment epithelial cells and intestinal epithelial cell lines. The results show that for HSEP2, the cell viability remained >80% for a peptide concentration range of 0–160 µg/mL. At a concentration of 200 µg/mL, the cell viability was >70%. HSEP3 too showed similar cell viabilities as HSEP2 for the assayed peptide concentrations (Fig. 10a and Supplementary Figure 6).

We also performed hemolytic assay on both peptides to check the effect of peptides on human RBCs. Figure 10b shows the results of the assay; we observed low hemolytic activity of the peptides toward RBCs. At a peptide concentration ranging from 0 to 200 μg/mL, both HSEP2 and HSEP3 showed <5% hemolytic activity. These results show that the peptides are relatively safe to use.

### Employing designed peptides for food preservation

In the previous sections, we have demonstrated that the designed peptides have high stability and antibacterial activity. These and similar peptides can in principle be employed for various applications, including topical medical applications, surface sterilization, food preservation, etc. Here, we focus on applications to food preservation.

For assessing the antibacterial efficacy of the peptides, we tested some selected peptides against a range of important spoilage bacteria some of which also cause food-borne illness. Four Gram-positive and three Gram-negative bacteria were selected for these assays (see “Methods”). The MIC values for all tested peptides are tabulated in Table 2. Comparing the MIC values of all peptides across different bacteria, we conclude that HSEP3 demonstrates the best antibacterial efficacy and inhibits a wider spectrum. For a maximum concentration up to 100 µg/mL, HSEP3 can kill 5 out of 7 bacterial species that were tested. Other peptides such as HSEP3b and HSEP3c also show reasonable activity (<=50 µg/mL) against important food pathogens such as *P. cartovorum, L. monocytogenes*, and *B. cereus*.

Further, we demonstrate the applicability of the best peptide in this study—HSEP3 as an antibacterial preservative. Since rice is a major staple food consumed all over the globe, we sought to test the efficacy of HSEP3 in preventing spoilage of cooked rice samples. Briefly, rice samples treated with HSEP3 were inoculated separately with *B. cereus*, *M. luteus*, and *L. monocytogenes* cells. The test samples were incubated for up to 6 days along with negative control samples (without HSEP3). Samples were aseptically obtained for testing every 24 h and the level of bacterial proliferation was monitored by (a) culturing samples on agar media to obtain the CFU values and (b) qualitative cell viability using resazurin dye (see “Methods” for details). We found no growth in the samples up to six days (Supplementary Figure 7). The control samples on the other hand, demonstrated a steady increase in CFU values over the assay duration.

Notably, Nisin—a peptide-based preservative used in food industry is relatively inadequate on accounts of both pH and temperature stability. It shows highest activity at pH 3 with rapid loss of activity as pH increases. The temperature stability of Nisin is also dependent on the ambient pH; it can be autoclaved without much loss of activity at pH 3, but at pH 7 it loses 90% of its activity, independent of the ambient temperature^6^. This severely limits applicability of Nisin. HSEP3 has comparatively better stability against high temperature and wide range of pH (Fig. 2 and Supplementary Table 2). Hence, HSEP3 is compatible with other methods of preservation such as thermal sterilization, acid regulation, etc. When HSEP3 and Nisin were compared for the antibacterial activity against *B. cereus*, HSEP3 showed better efficacy (Supplementary Table 1). We also compared HSEP3 and Nisin for their ability to inhibit *B. cereus* spore germination. HSEP3 demonstrated ~16-fold higher activity compared to the Nisin (Supplementary Table 2). To summarize, we demonstrate the potential of HSEP3 as an excellent alternative to current antimicrobial preservative. It demonstrates better stability and antimicrobial spectrum and therefore could be employed in cases where other preservatives such as Nisin are ineffective.

This work reports our attempt toward the rational design of antibacterial peptides—notably, with multiple properties like broad pH-resistance, exceptional thermostability, and anti-proteolytic activity—that enables their wider application to a variety of areas. Out of the 12 peptide variants designed in this study, 7 peptides show good MIC (<10 µg/mL) value against at least one bacterial sp., *M. luteus* (Tables 1 and 2). The best peptide design (HSEP3) shows reasonable antibacterial activity (<100 µg/mL) against five different bacterial species, while retaining stability against temperature, pH, and proteolysis. This demonstrates the advantage of rational design over a purely combinatorial approach. Typically, combinatorial methods require designing and screening several thousands to a million candidates to obtain good hits. Rational methods employing fundamental knowledge about various properties of the system can enable narrowing down to good hits within a few design iterations from a limited number of starting candidates. A combination of rational and combinatorial design strategies has been increasingly successful in designing proteins with novel functions^58–61^. A similar strategy can also be applied to design of peptides tailored for the application.

Our study focuses on the role of the *loop* in antibacterial action and the synergism between its trypsin inhibitory function and the membrane destabilizing action of the peptide *tail*. We show that (a) the peptides also inhibit intracellular trypsin and (b) both membrane destabilization and intracellular trypsin inhibitory activity of the peptide contribute to its overall antimicrobial activity. This means that it may be possible to redesign the ‘*loop*’ region against other essential intracellular proteins, which should further boost the inhibitory activity as well as the bactericidal spectrum of the peptides. While the *loop* serves as a determinant of intracellular target stability, the terminal region of the peptides can be redesigned to achieve maximal membrane destabilizing activity. Such strategies can be employed to expand the pool of functional antibacterial molecules. This finding is also of relevance to the design of synergistic combinations to obtain effective antibacterial peptide cocktails.

The insights obtained through the aforesaid studies are of broad importance to the design of antimicrobials for clinical and biomedical applications as well. In addition to the development of novel alternatives to current antimicrobials, synergistic combinations of new and existing peptides may pave way to more effective treatment of resistant infections. Moreover, by using combination of peptides with different modes of antibacterial action, the chance of developing bacterial resistance is also lowered. A case in point is the development of a superior version of the antibiotic—Vancomycin—that combines three different inhibition mechanisms, resulting in a molecule that has much better efficacy^62^.

Although in principle, the peptide designs may be applicable to a wide range of applications, a separate assessment of their efficacy is necessary for specific applications. For this study, we have tested the best peptide design (HSEP3) for its applicability as a food preservative and demonstrate that HSEP3 could be a potential alternative to current antibacterial preservatives like Nisin. However, one compelling argument against the application of trypsin inhibitor-based antimicrobial peptides could be its activity against digestive serine proteases. We reason that these peptides should be safe for human and animal consumption, if the inhibitor concentration required for effective food preservation is lower compared to the concentration that cause perceptible anti-nutritive effects during digestion—a concept that is similar to the Therapeutic Index used to assess systemic drugs. In relation to this, the studies on USFDA-approved soybean Bowman–Birk inhibitor concentrate (S-BBIC) (which is the same class of inhibitors as our peptides) are roughly indicative of the safety of trypsin inhibitor-based molecules for animal and human consumption. S-BBIC has been tested for its efficacy against various carcinogenic conditions in animal and human trials^63^. For these studies, a concentration of up to 10 mg/kg body weight of humans has been recommended as a safe dose. This value corresponds to roughly 500 µg/mL of peptide concentration which is more than 3-fold of the maximum MIC values obtained for our peptides (150 µg/mL). Although these values are rough estimates based on S-BBIC data, they suggest that HSEP3 could be an effective and safe candidate as a food preservative. However, animal studies are necessary to obtain a more reliable assessment of the potential of HSEP3.

Studies are currently underway to test the suitability of these peptides for other applications and develop better variants employing more robust design approaches. In addition, the molecular details underlying the difference in antibacterial efficacy against different bacteria need to be investigated in order to rationally develop peptides with broad-spectrum antibacterial activity.

## Methods

### Peptide and microbial strains

The peptides used in this study as well as the FITC (fluorescein isothiocyanate) labeled peptides were synthesized by Pepmic Co., Ltd (Suzhou, China). The purity of the peptides (>95%) was assessed by reverse-phase high-performance liquid chromatography (HPLC) and the molecular weight was confirmed by Electrospray Ionization Mass Spectrometry (ESI-MS). Mueller-Hinton (MH) broth and Luria Bertani (LB) broth media were procured from Hi Media, India. Ethanol, Triton X-100, and Glutaraldehyde were procured from Merck, Germany. Trypsin and BAPNA (N-alpha-benzoyl-DL-arginine-p-nitroanilide) were procured from Sigma-Aldrich. WST-I was procured from Roche, Indianapolis, IN. All other chemicals used were of the highest purity available.

Four Gram-positive bacteria: *Listeria monocytogenes* ATCC13932*, Bacillus cereus* ATCC11778*, Staphylococcus aureus* ATCC12900*,* and *Micrococcus luteus* ATCC4698 and three Gram-negative bacteria: *Escherichia coli* ATCC11775, *Pectobacterium carotovorum* MCC2112, and *Salmonella typhimurium* ATCC9844 were used in this study. These strains were stored at −80 °C in 25% (v/v) glycerol.

### Antimicrobial assays

Antimicrobial assays against various bacterial cultures were done using the microdilution broth assay according to the Clinical and Laboratory Standards Institute (CLSI)^64^ with some modifications. Mueller-Hinton broth was used to dilute the peptide stock and the bacterial inoculum. Inoculum was prepared from the mid-logarithmic phase culture. Each well of the microtiter plates received aliquots of 100 µL of the media containing different concentrations of peptide ranging from 0.3 to 300 µg/mL. The final concentration of bacteria in the wells was 5 × 10^5^ Colony Forming Units (CFU) per mL. Ampicillin was used as positive control. For negative control, ultrapure water was used instead of peptides. Microtiter plates were incubated at 37 °C for 5–6 h with continuous shaking at 130 rpm (*M. luteus* cells were incubated for 7–8 h). The incubation time used for these assays was initially optimized using a 20-h incubation period. Minimum Inhibitory Concentration (MIC) was determined by visually observing the color change after adding resazurin dye into each well at a final concentration of 37 µg/100 µL. Here, MIC is defined as the lowest dilution of peptide that completely inhibited the growth of the organism. To determine the MIC combination of peptides, HSEP3 was mixed with HSEP2-∆HR or HSEP2-∆HR,∆K8 in 1:3, 2:2, 3:1 (HSEP3: HSEP2-∆HR/HSEP2-∆HR,∆K8) ratios (w/w) and antimicrobial assays were carried out for the determination of MIC values. MIC assays were performed as three independent experiments in triplicates.

### Propidium iodide (PI) uptake assay

*B. cereus* and *M. luteus* were grown in MH broth to logarithmic phase of growth and then diluted to OD_600_ ~ 0.25. Serial dilutions of the antimicrobial peptides (final concentrations, 0–100 μg/mL) were added to the wells of a black-walled microplate. Hundred microliters of the bacterial suspension containing PI (final concentration, 10 μM) was added to the wells. The emitted fluorescence was measured at excitation and emission wavelengths of 585 and 620 nm, respectively, for 2 h using a multimode plate reader (Varioskan Flash, Thermo Scientific, USA). The percentage of membrane permeabilization was calculated as the percent of fluorescent intensity of peptide-treated samples with respect to fluorescence intensity of PI-loaded, peptide untreated samples. PI uptake assays were performed as three independent experiments in triplicates.

### Membrane permeability assay

As a qualitative study, membrane permeability and bacterial viability were analyzed using LIVE/DEAD BacLight bacterial viability assay kit for microscopy [L7007, from Invitrogen, this is a mixture of stains: SYTO 9 (for live cells) and PI (for dead cells)] according to manufacturer’s instructions. Briefly, 3 μL of the staining mixture was added to 1 mL of the bacterial cells previously treated with different concentrations of HSEP3 (6.25 and 12.5 μg/mL for *B. cereus*; 0.7 and 1.25 μg/mL for *M. luteus*; 85 μg/mL for *P. caratovorum*) for 1 h, and saline-treated cells were kept as control. The samples were incubated for 15 min in dark at room temperature and 5 μL of this sample was trapped in between coverslip and glass slide. The slide was viewed under a confocal fluorescence microscope (LSM700, Carl Zeiss, Germany), using ×63 objective using the following settings: excitation/emission of 480/500 nm and 490/635 nm for SYTO 9 and PI, respectively.

### Scanning electron microscopy (SEM)

*Bacillus cereus* and *Micrococcus luteus* cells were grown in LB broth at 37 °C to mid-log phase under continuous shaking at 180 rpm. Cells were harvested by centrifugation at 5500 rpm for 5 min, washed thrice with 10 mM PBS, diluted 1 × 10^8^ CFU/mL with PBS. Cells were incubated with 1X MIC of peptide in a 500 µL reaction for 1 h. Control cells were incubated without peptides. After incubation cells were harvested by centrifugation at 8000 rpm for 5 min, washed thrice with PBS, fixed with 2.5% (w/v) glutaraldehyde at room temperature for 4 h, followed by washing twice with PBS. The cells were dehydrated for 10 min with a graded ethanol series (25, 50, 75, 95, and 100%). Pellet was dissolved in 100% ethanol and dried at room temperature. The samples were mounted on the specimen holder and sputter-coated with gold. Samples were finally transferred to electron microscope (LEO 435 VP, USA) for imaging.

### Transmission electron microscopy (TEM)

*B. cereus* cells were grown and incubated with AMPs as described above for SEM sample preparation. Cell pellets were obtained from 10 mL of each control or treated cell suspension and fixed with 2.5% buffered glutaraldehyde. The cells were post-fixed with 1% osmium tetroxide for 2 h at room temperature. This was followed by dehydration in a graded ethanol series (70%, 80%) for 1 h and ‘en-bloc’ staining with 1% uranyl acetate in 95% ethanol for 1 h. The final dehydration was carried out in absolute alcohol for 30 min at 4 °C. The clearing was done with propylene oxide at room temperature. The cells were then processed for infiltration; wherein propylene oxide was replaced with liquid resin araldite CY212. Infiltration was carried out on a rotator in two steps: a mixture of propylene oxide and araldite (1:1) overnight and two changes in pure araldite for 2 h at room temperature. Cells were allowed to polymerize in an oven at 60 °C for 48 h. Finally, 40–60-nm-thick sections were collected on 300 mesh copper grids, stained with lead citrate, and were viewed under the transmission electron microscope (FEI, TECNAI G2 Spirit BioTwin, Netherlands).

### Confocal laser-scanning microscopy

*B. cereus* and *M. luteus* cells in mid-logarithmic phase were harvested by centrifugation, washed three times with 10 mM PBS, pH 7.2. 1 × 10^7^ CFU/mL cells were incubated with fluorescence (FITC) labeled peptide at 1X MIC and 2X MIC concentration at 37 °C for 1 h. After 1 h, cells were pelleted down and washed three times with PBS and spotted on a glass slide, and observed under a confocal microscope (LSM700, Carl Zeiss, Germany) with a ×63 objective. Fluorescent images were obtained with a 488 nm band-pass filter for excitation of FITC. Similarly, for PI fluorescence images, the peptide-treated cells were incubated with PI (1.3 µg/mL) for 20 min in dark and were observed under confocal microscope.

### Hemolytic assay

The hemolytic activity of the peptide was evaluated using human red blood cells (hRBCs). Erythrocytes were separated from 1 mL of blood by centrifugation at 1500 rpm for 10 min. Collected hRBCs were washed three times with PBS, diluted to 4% (v/v) in PBS. Hundred microliters of the hRBCs having peptides ranging from 4 to 200 µg/mL as added into 96-well microtiter plate. The plates were incubated for 1 h at 37 °C without agitation and centrifuged at 1500 rpm for 5 min. Aliquots (100 μL) of the supernatant were transferred to 96-well plates and absorbance was measured at 414 nm. PBS and 1% Triton X-100 were used as control for 0% and 100% hemolysis, respectively. Percentage of hemolysis was calculated as (*A*_T_ − *A*_C_)/(_AX_ − *A*_C_) × 100; where *A*_T_ is the experimental absorbance of treated supernatants, *A*_C_ is the control absorbance of PBS-treated cell supernatant, and _AX_ is the absorbance of 0.1% (v/v) Triton X-100 lysed cells. Data presented are averages and standard error from three biological replicate experiments.

### Cytotoxicity assay

Human Intestinal Epithelial Cells (HIEC-6, ATCC CRL-3266) were grown in the base medium OptiMEM-1 Reduced Serum Medium containing the following components: 20 mM HEPES, 10 mM GlutaMAX, 10 ng/mL epidermal growth factor (EGF), and fetal bovine serum (FBS) to a final concentration of 4%. Cells were incubated at 37 °C with 5% CO_2_ and 95% air. 10,000 cells were transferred to wells of a 96-well plate at a concentration of 10,000 cells per well. Cells were treated with 100, 250, 500, 750, and 1000 μg of HSEP3 and incubated at 37 °C with 5% CO_2_. The percentage viability of cells was determined by using the MTT assay. The media was removed from the wells after 12 h of incubation. Ten microliters of 5 mg/mL solution of MTT was added to each well and incubated for 4 h. The MTT-containing medium was removed and the formazan crystals were dissolved in DMSO. The absorbance was measured at 530 nm.

Cytotoxicity against adult retinal pigmented epithelial (ARPE-19, ATCC CRL2302) cells was measured using WST assay. ARPE-19 cells growing in log phase were seeded into 96-well cell-culture plates at 4 × 10^4^ the cells were incubated at 37 °C for 24 h under 5% CO_2_. Peptide is added with final concentrations of 4–200 µg/mL in Dulbecco’s modified Eagle medium (DMEM/F12). Nutrient mixture media for the treatment group, whereas for the negative control group, media alone was added. The cells were incubated for 16 h at 37 °C under 5% CO_2_. Ten microliters of WST-1 reagent was added into each well. Plate was incubated at 37 °C for 2 h. Color intensity was measured at 450 nm. Cytotoxicity data presented are averages and standard error from three biological replicate experiments.

### Trypsin inhibition studies

The amidase activity of trypsin and its inhibition was assayed using the chromogenic substrate BAPNA at pH 8.2 in 0.05 M Tris-HCl containing 0.02 M CaCl_2_ at 37 °C. The assay reaction contained 50 µL of trypsin solution (40–50 μg of trypsin in 1 mM HCl), 50 µL of water, and 125 µL of the substrate. The reaction was carried out at 37 °C for 10 min and stopped by addition of 0.25 mL of 30% acetic acid. Absorbance of the liberated p-nitroaniline was measured at 410 nm against an appropriate blank in which the reaction was arrested by adding 30% acetic acid prior to BAPNA addition. The trypsin solution was incubated with an aliquot of inhibitor for 10 min at 37 °C and reaction started by the addition of 125 µL substrate and incubated at 37 °C for 10 min. The reaction was arrested by the addition of 30% acetic acid and the residual trypsin activity was measured by recording the absorbance at 410 nm. One unit of trypsin enzyme activity is defined as an increase in the absorbance of 0.01 at 410 nm under the assay conditions. The effect of varying substrate concentration (BAPNA) on bovine trypsin in the presence of fixed concentrations of peptides was studied. The inhibition constants (*K*_i_) were evaluated using the double reciprocal and Dixon plot of the data^65,66^. Data presented are averages and standard error calculated from two biological replicate experiments.

### Thermostability and pH-stability studies

Thermostability was measured by the determination of trypsin inhibition activity of the peptides after incubation for 30, 60, 90, 120, 150, and 180 min at 95 °C. For pH stability, peptides were dissolved in 50 mM buffers of pH 2.5, 5, 9 and incubated for 2 h at room temperature and trypsin inhibition activity of the peptides was assayed using the BAPNA method described earlier. Data presented are averages and standard error calculated from two biological replicate experiments.

### Effect of heating and autoclaving on antibacterial activity

To determine the stability of HSEP3 at higher temperatures, 20 μL of HSEP3 (4 mg/mL) and 20 μL of Nisin in a 0.5 mL tube were autoclaved (for moist heat) at 121 °C for 20 min or heated at 95 °C for 30 min (for dry heat) and cooled to room temperature. Mixture was then used to test the antibacterial activity as previously described. Experiments were repeated in LB media to study the effect of complex media on antibacterial activity. All the experiments were repeated at least two times.

### Spore germination studies

*B. cereus* spores were used to test the effect of peptides on spore germination. Spores were prepared by inoculating 3 mL MH media with *B. cereus* cells and incubating at 30 °C at 180 rpm for 12 h. 2.5 mL of the culture was inoculated into a 250 mL MH media and incubated at 30 °C for 72 h. Furthermore, culture was temperature-treated at 80 °C for 30 min to eliminate the vegetative cells. Spore suspension was stored at 4 °C until further use. Two microliters of spore suspension was used to test the effect of HSEP3 and Nisin on spore germination using resazurin. All experiments were repeated at least twice.

### Molecular dynamics of lipid bilayer-peptide-water system

For the simulations, HSEP3 structure was modeled on the available crystal structure of SFTI bound to bovine trypsin (PDB ID: 1SFI) using Modeller v9.11^67^. Only the disulfide-restricted inhibitory *loop* was modeled on the structure, while the flexible *tail* segments were optimized using Modeller. The top-scoring (DOPE score) structure obtained was simulated for 50 ns using isotropic pressure (1 bar; Parrinello–Rahman barostat) and temperature coupling (298 K; velocity rescaling^68,69^ to obtain a structural ensemble of the peptide in aqueous environment. GROMACS-5.1.2^70^ package was used for performing the simulation studies. All simulations were performed using CHARMM36 parameter set. Peptide structures were clustered using the gromos clustering method and a representative structure from the top cluster was used for membrane-peptide-water simulations^71^.

The lipid bilayers were constructed using the Membrane Bilayer Builder available at the CHARMM-GUI webserver^72^. We chose to construct POPE-POPG bilayer for these studies. The lipid bilayer consists of POPE:POPG in 3:1 ratio, in accordance with the experimentally observed fraction of POPG in bacterial membrane (~23% POPG in bacterial membrane). All simulations were performed on GROMACS-5.1.2 using the CHARMM36 parameter set^73^. We chose the CHARMM36 parameters for these simulations because compared to other available lipid parameters, CHARMM parameters have been shown to model POPG correctly^74^. Initially, the membrane bilayer was equilibrated for a total of 50 ns. For membrane-peptide simulations, coordinates for the lipid bilayer were extracted from the final frame obtained from the 40 ns membrane-water simulations.

Three lipid bilayers systems were constructed with POPE:POPG in the ratio of 3:1 and containing 200 (POPE:150, POPG:50), 400 (POPE:300, POPG:100), and 800 (POPE:600, POPG:200) lipids, respectively. All three systems were solvated using a value of 50 as the hydration number (i.e., number of water molecules per molecule of lipid) and adequate number of Na^+^ ions were added for neutralizing the system. The systems were minimized, and thereafter, equilibrated with position restraints on the phosphorus atoms, and dihedral restraints on the glycerol backbone and unsaturated carbon pairs. The equilibration was performed in multiple steps with decreasing force constant values used for the restraints. Equilibration steps (100 ps each) involved two steps of NVT using the Berendsen thermostat^75^ for temperature coupling (*T* = 315 K). A temperature of 315 K was chosen for these simulations which is greater than the phase transition temperatures of POPE [299 K] and POPG [268 K]. This was followed by four steps of NPT (total 8 ns) equilibration using the Berendsen thermostat and barostat for temperature and semi-isotropic pressure coupling wherein the restraints were gradually reduced. Finally, all systems were simulated (without restraints) in NPT ensemble (*T* = 315 K; *P* = 1 bar) for 40 ns each, and the final frames of the trajectories were used for final production simulations with peptides. For production runs, we used Nose-Hoover^76–78^ thermostat for temperature coupling (coupling constant = 1.0 ps) and Parrinello–Rahman barostat for semi-isotropic pressure coupling (coupling constant = 5.0 ps). A leap-frog integrator^79^ was used with a time-step of 2 fs for all runs with constraints on all hydrogen bonds using the P-LINCS algorithm^70^. The long-range Coulomb interactions were treated using Smooth Particle Mesh Ewald^80,81^ and Van-der-Waals interactions were treated using a twin-range cut-off scheme. Van-der-Waals interactions were truncated at 1.0 nm with the potential smoothly shifted to zero at 1.2 nm.

For constructing the peptide-membrane system, eight HSEP3 peptide molecules (ratio of lipid:peptide = 100:2) were randomly placed on one side of the membrane bilayer at a distance of ~5 nm from the bilayer. This system was placed in a simulation box of size ~11 × 11 × 11 nm^3^, solvated with water (~27,000 molecules), and appropriate counter-ions were added to neutralize the system charge. The system was minimized and equilibrated similar to the protocol used of membrane simulations (see the previous paragraph). Since the membrane and peptide coordinates used for constructing the membrane-peptide systems are already equilibrated structures, we used a shorter three-step protocol here to obtain equilibrated systems for production runs; (a) minimization step with position restraints on peptide and lipids, (b) NVT equilibration step with position restraints on peptide and lipids, and (c) NPT equilibration with position restraints on lipids alone—in total 4 ns of equilibration simulation. In the course of our preliminary production simulations, we observed that once few peptide molecules adhere to the membrane surface, other charged peptides experience high repulsive forces that do not allow these other peptides to interact with the membrane. Many of these non-interacting peptides quickly move away and re-enter the simulation box from the other side of the membrane and adhere to the lower leaflet. Such a behavior has been observed previously by Li et al.^52^. To circumvent this problem, we have used a similar approach as employed by Li et al.^52^; we first performed pulling simulations on the equilibrated peptide-membrane system by applying a weak pulling force (k = 100 kJ/mol/nm^2^) between the center-of-mass of individual peptides and the membrane. To avoid any unwanted movement of lipid molecules during the pulling simulations, we applied position restraints (*k* = 500 kJ/mol/nm^2^) on the heavy atoms of all lipid molecules. The above steps allowed all the peptides to bind to the upper leaflet. Subsequently, this system was equilibrated for additional 2 ns before commencing the final 1.5 µs production simulation that was used for studying membrane-peptide interactions. The simulation was performed with the same run-parameters as used for membrane-water simulations. TIP3P explicit water model was used for all simulations. Three independent simulations were run, each with different initial peptide positions and orientations, as well as randomly assigned initial atomic velocities for the given temperature, based on Maxwell-Boltzmann distribution. An additional 300 ns control simulation (membrane only) was also run for comparison of equilibrium properties with those calculated for peptide-bound membrane simulations.

All visualization, structural analyses, and rendering were done using Chimera^82^ and VMD^83^. The membrane properties were calculated and plotted using analyses programs included in the GROMACS package and custom python scripts. An in-house modified version of GridMAT-MD program was used for calculating and plotting area-per-lipid and bilayer thickness over the full trajectory^84^.

For membrane-only simulations, the final 100 ns of the trajectory were analyzed to obtain the average area-per-lipid and thickness value for the bilayer. For the peptide-membrane simulations, the calculations were performed on the final 700 ns of the trajectory. All graphs and plots were produced and rendered using Grace.

### Testing efficacy as a food preservative

Cooked rice samples (1 g) were sterilized and aseptically transferred into polypropylene tubes (12 × 5 mL) and mixed thoroughly with an appropriate concentration of aqueous solution of HSEP3 (excluding controls). All 12 tubes were inoculated with 1 × 10^5^ CFU/g load (from broth cultures after 24-h incubation) of testing microorganisms. Among 12 tubes, three tubes were inoculated with *L. monocytogenes*, three with *B. cereus* and three with *M. luteus*. One tube was maintained as control for each microorganism without adding the peptide. All the tubes were sealed and stored at room temperature. At various time intervals (0, 24, 48, 72, 96, 120, 144 h), content from each tube was drawn aseptically and plated on the media by using spread plate method. Plates were incubated at 37 °C and bacterial counts were calculated at each time point.

### Reporting summary

Further information on research design is available in the Nature Research Reporting Summary linked to this article.

## Supplementary information

Reporting Summary

Supplementary Information

## Data Availability

The data that support the findings of this study are in supplemental data including both experimental data and computational data. All other data are available from the corresponding author on reasonable request.
