# Supplementary material for: Rational design of hyperstable antibacterial peptides for food preservation
Source: NPJ Sci Food. 2021 Sep 1;5:26. doi: 10.1038/s41538-021-00109-z (PMC8410836; doi:10.1038/s41538-021-00109-z)
Supplement: Supplementary file 1 — Reporting Summary [file 41538_2021_109_MOESM1_ESM.pdf]

nature research

Corresponding author(s): Balaji Prakash
Last updated by author(s): Mar 6, 2021

Reporting Summary

Nature Research wishes to improve the reproducibility of the work that we publish. This form provides structure for consistency and transparency in reporting. For further information on Nature Research policies, see our Editorial Policies and the Editorial Policy Checklist.

Statistics

For all statistical analyses, confirm that the following items are present in the figure legend, table legend, main text, or Methods section.

n/a	Confirmed	
x		The exact sample size (*n*) for each experimental group/condition, given as a discrete number and unit of measurement
	x	A statement on whether measurements were taken from distinct samples or whether the same sample was measured repeatedly
x		The statistical test(s) used AND whether they are one- or two-sided<br>*Only common tests should be described solely by name; describe more complex techniques in the Methods section.*
x		A description of all covariates tested
x		A description of any assumptions or corrections, such as tests of normality and adjustment for multiple comparisons
x		A full description of the statistical parameters including central tendency (e.g. means) or other basic estimates (e.g. regression coefficient) AND variation (e.g. standard deviation) or associated estimates of uncertainty (e.g. confidence intervals)
x		For null hypothesis testing, the test statistic (e.g. *F*, *t*, *r*) with confidence intervals, effect sizes, degrees of freedom and *P* value noted<br>*Give P values as exact values whenever suitable.*
x		For Bayesian analysis, information on the choice of priors and Markov chain Monte Carlo settings
x		For hierarchical and complex designs, identification of the appropriate level for tests and full reporting of outcomes
x		Estimates of effect sizes (e.g. Cohen's *d*, Pearson's *r*), indicating how they were calculated

Our web collection on statistics for biologists contains articles on many of the points above.

Software and code

Policy information about availability of computer code

Data collection	No commercial or open source databases used
Data analysis	The data analysis methods are all clearly described in the manuscript. Xmgrace and Graphpad prism 8 was used to plot data.

For manuscripts utilizing custom algorithms or software that are central to the research but not yet described in published literature, software must be made available to editors and reviewers. We strongly encourage code deposition in a community repository (e.g. GitHub). See the Nature Research guidelines for submitting code & software for further information.

Data

Policy information about availability of data

All manuscripts must include a data availability statement. This statement should provide the following information, where applicable:

- Accession codes, unique identifiers, or web links for publicly available datasets
- A list of figures that have associated raw data
- A description of any restrictions on data availability

The data that support the findings of this study are in supplemental data including both experimental data and computational data. All other data are available from the corresponding author on reasonable request.

Field-specific reporting

Life sciences study design

All studies must disclose on these points even when the disclosure is negative.

Sample size	Not relevant
Data exclusions	No values were excluded from any analysis
Replication	experiments were replicated independently at least two times. All attempts of replication were successful.
Randomization	Bacteria were divided in to ´control´ (without peptide treatment) and ´treated´ (with peptide treatment) groups.
Blinding	Blinding was not required for the experiments associated with this study

Reporting for specific materials, systems and methods

We require information from authors about some types of materials, experimental systems and methods used in many studies. Here, indicate whether each material, system or method listed is relevant to your study. If you are not sure if a list item applies to your research, read the appropriate section before selecting a response.

Materials & experimental systems

n/a	Involved in the study	
☒	☐	Antibodies
☐	☒	Eukaryotic cell lines
☒	☐	Palaeontology and archaeology
☒	☐	Animals and other organisms
☒	☐	Human research participants
☒	☐	Clinical data
☒	☐	Dual use research of concern

Methods

n/a	Involved in the study	
☒	☐	ChIP-seq
☒	☐	Flow cytometry
☒	☐	MRI-based neuroimaging

Eukaryotic cell lines

Policy information about cell lines

Cell line source(s)	Human Intestinal Epithelial Cells (HIEC-6, ATCC CRL-3266), Adult Retinal Pigmented Epithelial (ARPE-19, ATCC CRL2302)
Authentication	both the cell lines were obtained from ATCC. Original cell stocks were maintained in liquid N2 and cells were not used after 20 passages.
Mycoplasma contamination	Cell lines were negative to mycoplasma (tested and verified by ATCC)
Commonly misidentified lines (See ICLAC register)	No commonly misidentified cell lines used
