# Supplementary material for: Rational design of hyperstable antibacterial peptides for food preservation
Source: NPJ Sci Food. 2021 Sep 1;5:26. doi: 10.1038/s41538-021-00109-z (PMC8410836; doi:10.1038/s41538-021-00109-z)
Supplement: Supplementary file 2 — Supplementary Information [file 41538_2021_109_MOESM2_ESM.pdf]

Rational design of hyperstable antibacterial peptides for food preservation.

Yashavantha L. Vishweshwaraiah[†1], Abhishek Acharya[†1], Vinayak Hegde[1,2], Balaji Prakash[1,3]*

† These authors contributed equally to the work.

[1] Department of Molecular Nutrition, CSIR-Central Food Technological Research Institute, Mysore 570020, India

[2] Academy of Scientific and Innovative Research, Ghaziabad, Uttar Pradesh, India-201002.

[3] Division of Biological and Life Sciences, School of Arts and Sciences, Ahmedabad University, Navrangpura, Ahmedabad – 380009. Gujarat, India.

*To whom correspondence should be addressed: Telephone: 91-821-251-4192. Email: bprakashiit@gmail.com

Supplementary Figures

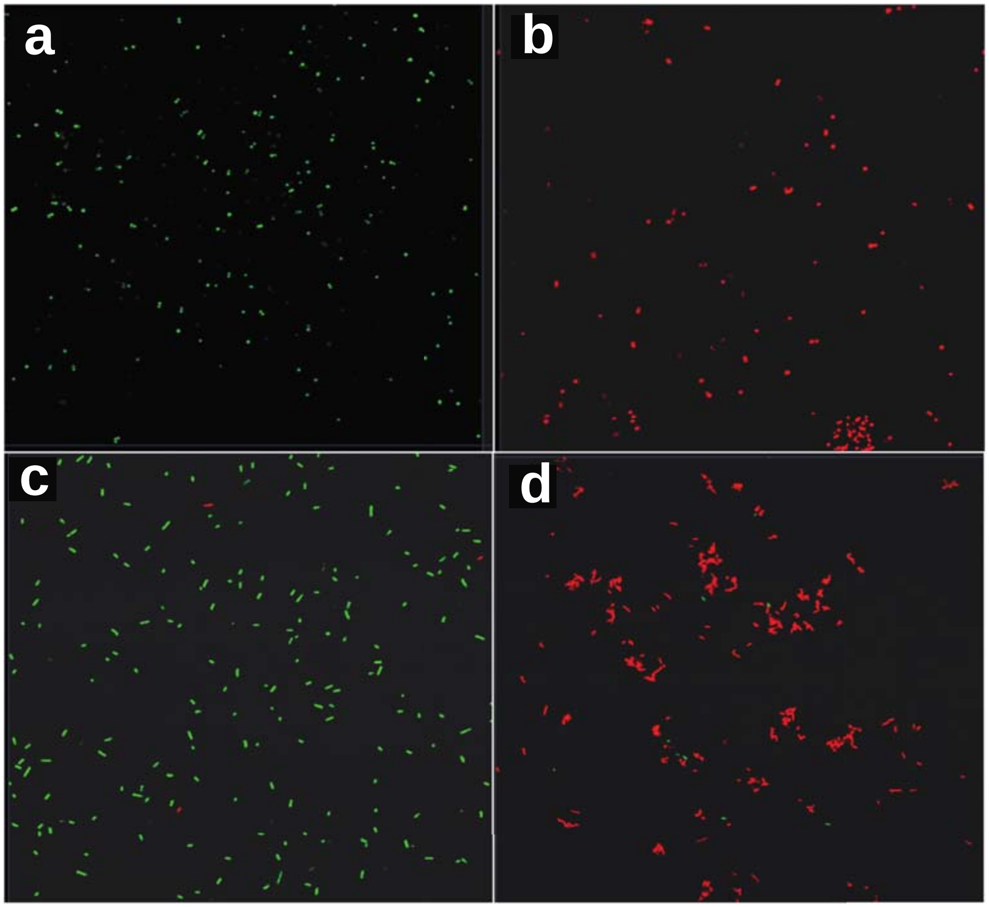

Supplementary Figure 1. Live-dead fluorescence staining of *M. luteus* and *P. carotovorum* demonstrating the antibacterial effect of HSEP3 peptide. Panel (**a**) and (**c**) denote control samples (no peptide added) of *M. luteus* and *P. carotovorum* cells, respectively. Panel (**b**) and (**d**) denote HSEP3-treated (at 1X MIC concentration) cells of *M. luteus*; and *P. carotovorum*, respectively. All samples have been treated with both SYTO-9 (indicates live cells) and propidium iodide (indicates dead cells) prior to imaging.

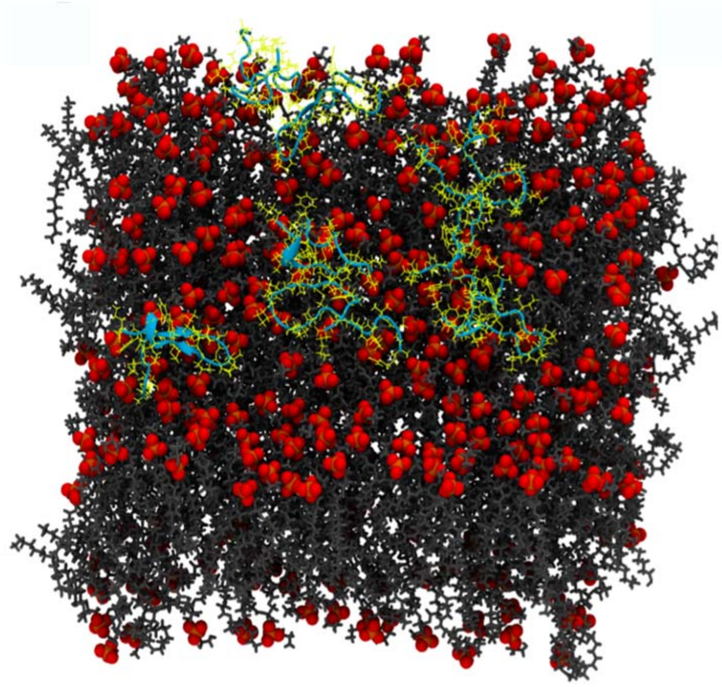

Supplementary Figure 2. Starting frame for the peptide membrane simulations. HSEP3 peptides simulated with 400-lipid POPE-POPG [3:1] membrane. Peptides are shown in blue bound to the top of the bilayer.

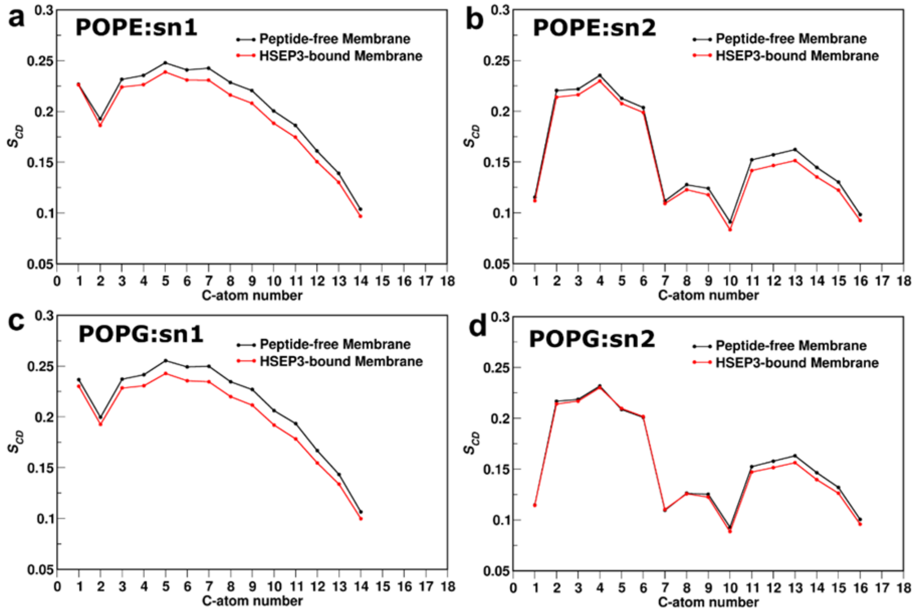

Supplementary Figure 3. Acyl-chain deuterium order parameter (S_{CD}) profiles calculated from peptide-free and peptide-bound membrane simulations. Panel (**a**) and (**c**) show the order parameter profile for the *sn1*-chain (i.e. palmitoyl) of POPE and POPG, respectively, while panel (**b**) and (**d**) plot the profile for *sn2*-chain (i.e. oleoyl) of POPE and POPG, respectively. The chain parameters for peptide-free simulations have been calculated from the final 50 ns of the trajectory. Whereas for peptide-bound simulations, the last 100 ns of the trajectory were used.

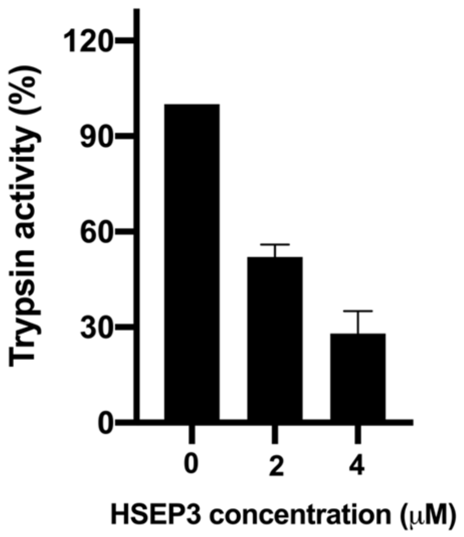

Supplementary Figure 4. Effect of HSEP3 on the intracellular trypsin activity. The activity is represented as percentage trypsin activity relative to the intracellular trypsin activity of the cell lysate without HSEP3. Data represent mean ± s.e.m. of two biological replicates.

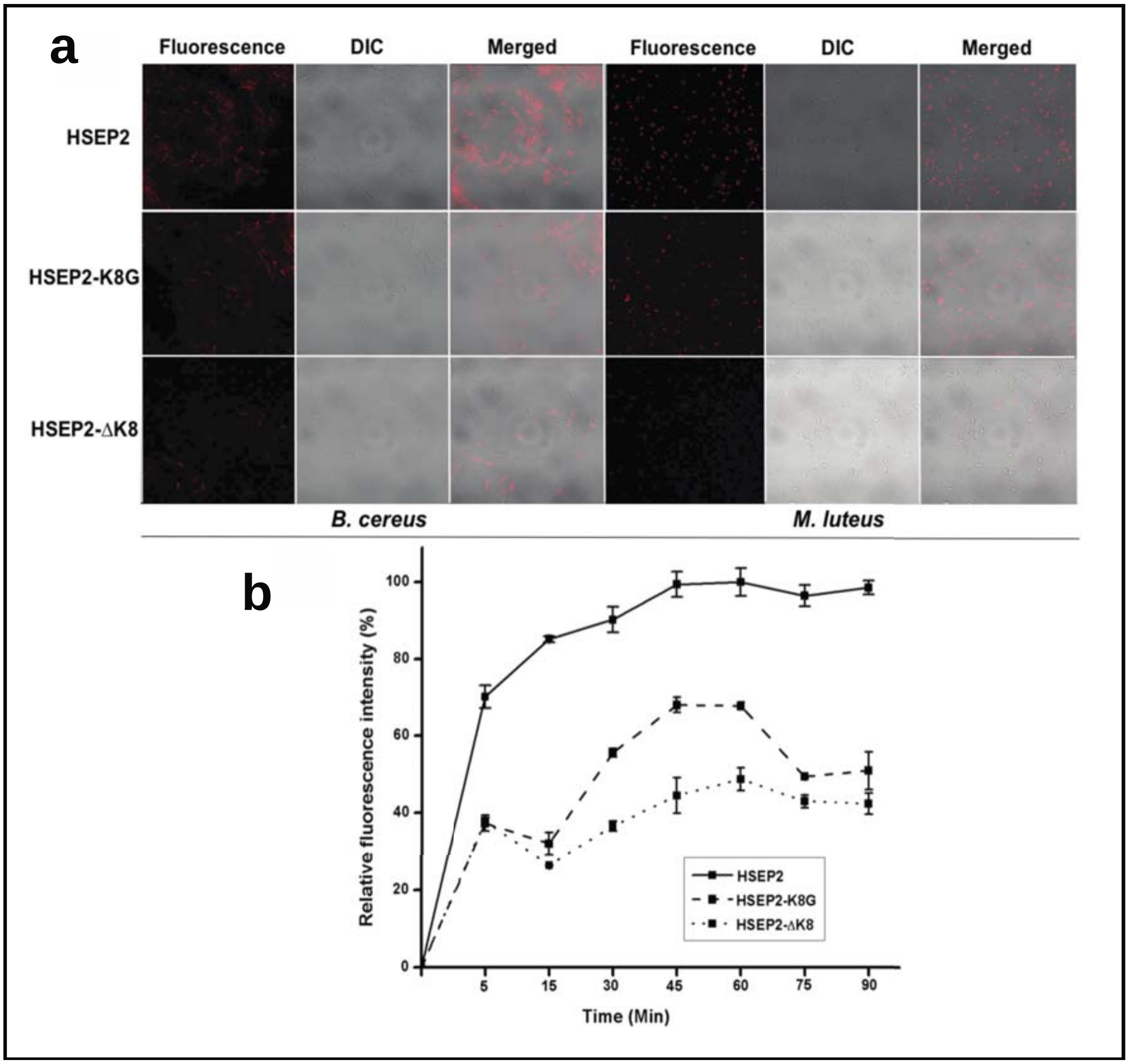

Supplementary Figure 5. Membrane permeabilization in *B. cereus* and *M. luteus* cells treated with HSEP2 and HSEP2-mutant peptides. (**a**) Confocal images of *B. cereus* and *M. luteus* cells incubated with HSEP2, HSEP2-K8G, HSEP2-ΔK8 and stained with propidium iodide. (**b**) Permeabilization of the cytoplasmic membrane of *M. luteus* by HSEP2, HSEP2-K8G and HSEP2-ΔK8 at 2X MIC, indicated by percent of propidium iodide fluorescence. Data represent mean ± s.e.m. of three biological replicates.

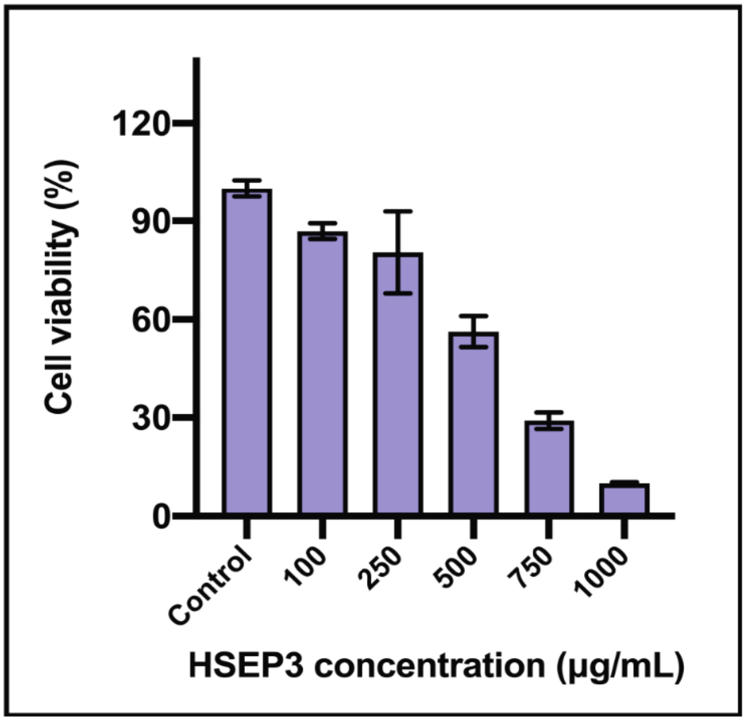

Supplementary Figure 6. Cytotoxicity of HSEP3 on human intestinal epithelial cells. Data represent mean ± s.e.m. of three biological replicates.

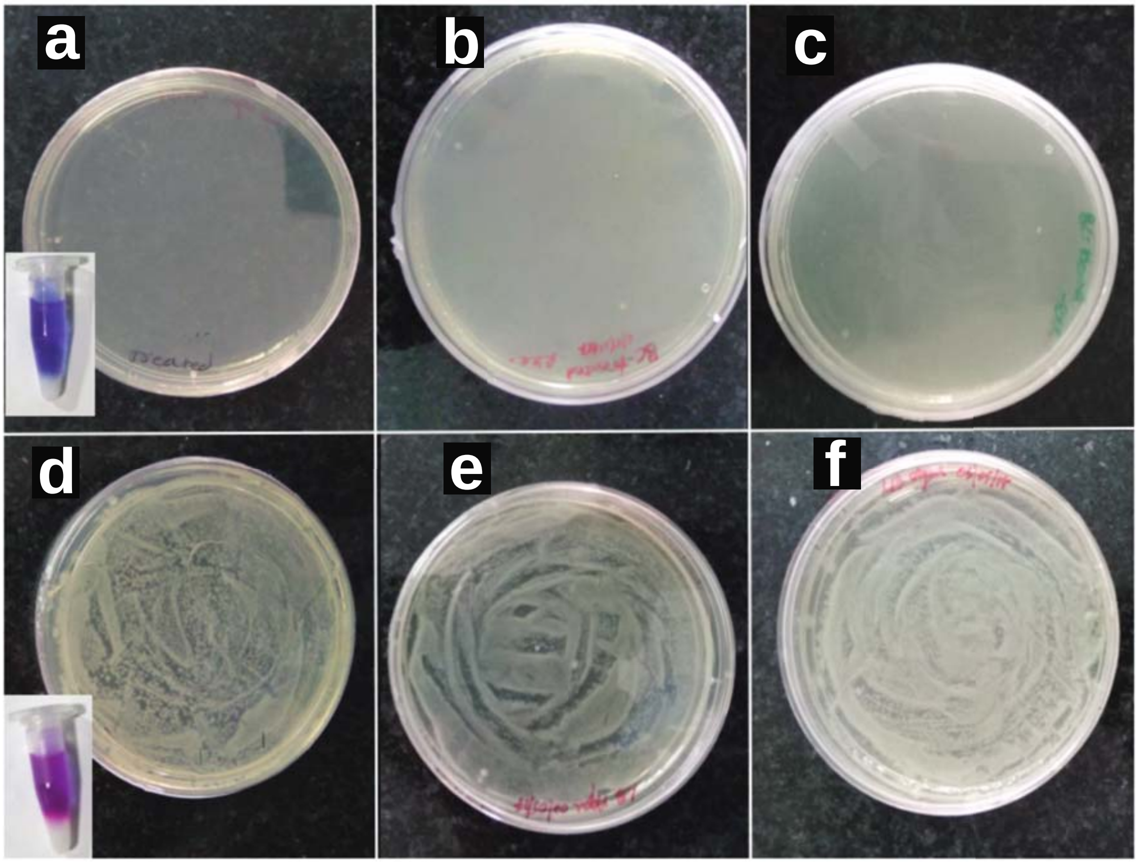

Supplementary Figure 7. Effect of HSEP3 peptide on the growth of *B. cereus* cells on cooked rice samples, as determined by plating on LB agar media. Panel (**a**), (**b**) and (**c**) show results for HSEP3-treated [2X MIC] rice samples, spiked with *B. cereus* cells, evaluated at 2, 4 and 6 days after inoculation respectively. Panel (**d**), (**e**) and (**f**) represent peptide untreated *B. cereus* cells evaluated in the same conditions. The inset images depict the resazurin dye test for the qualitative determination of viability of *B. cereus* cells in rice samples. The pink colored product indicates bacterial growth.

Supplementary Tables

Supplementary Table 1. Membrane bilayer properties calculated from (peptide-free) membrane simulations.

System	No. of Lipids (N_L)	Constituents	Temp [K]	APL (A_L) [Å^2]*	Bilayer Thickness (D_{P-P}) [nm]**	Isothermal Area Compressibility Modulus (K_A) [mN/m]#
200-lipid	200 (100/leaflet)	POPE: 150 POPG: 50	315	59.62±1.01	4.12±0.06	253.25
400-lipid	400 (200/leaflet)	POPE: 300 POPG: 100	315	59.43±0.71	4.13±0.04	252.0
800-lipid	800 (400/leaflet)	POPE: 600 POPG: 100	315	59.76±0.49	4.12±0.03	23.1

* Previously reported value for area-per-lipid of POPE-POPG bilayer: 58.3 Å^2 [Hong et al. 2014]; 61.5 Å^2 [Rog et al. 2003].
** Previously reported values for bilayer thickness (D_{P-P}): 4.01±0.01 nm [Pandit et al. 2012].
# The experimental range for Isothermal Area Compressibility Modulus values for bilayer is 180-330 mN/m. Previously reported value of K_A for POPE-POPG simulations is 0.25±0.04 N/m [Pandit et al 2012].

Supplementary Table 2. MICs of HSEP3 and Nisin against *B. cereus*.

Antibacterial activity against B. cereus		Inhibition of B. cereus spore germination		Effect of steam heat on antibacterial activity (in complex media)		Effect of steam heat on antibacterial activity (in MH media)		Effect of dry heat on antibacterial activity	
**HSEP3**	**Nisin**	**HSEP3**	**Nisin**	**HSEP3**	**Nisin**	**HSEP3**	**Nisin**	**HSEP3**	**Nisin**
12.5	50	12.5	200	50	ND	12.5	100	12.5	100

All values are in µg/mL. ND not detected

Supplementary References

1. Hong, C., Tieleman, D. P. & Wang, Y. Microsecond molecular dynamics simulations of lipid mixing. *Langmuir* **30**, 11993–12001 (2014).

2. T, R., K, M. & M, P.-G. Molecular dynamics simulations of charged and neutral lipid bilayers: treatment of electrostatic interactions. *Acta Biochim. Pol.* **50**, 789–798 (2003).

3. Pandit, K. R. & Klauda, J. B. Membrane models of *E. coli* containing cyclic moieties in the

aliphatic lipid chain. *Biochim. Biophys. Acta* **-** *Biomembr.* **1818**, 1205–1210 (2012).
